# Novel chitosan and bacterial cellulose biocomposites tailored with polymeric nanoparticles for modern wound dressing development

**DOI:** 10.1080/10717544.2021.1977423

**Published:** 2021-09-22

**Authors:** Paul-Octavian Stanescu, Ionut-Cristian Radu, Rebeca Leu Alexa, Ariana Hudita, Eugenia Tanasa, Jana Ghitman, Oana Stoian, Aristidis Tsatsakis, Octav Ginghina, Catalin Zaharia, Mikhail Shtilman, Yaroslav Mezhuev, Bianca Galateanu

**Affiliations:** a Advanced Polymer Materials Group, University Politehnica of Bucharest, Bucharest, Romania; b Department of Biochemistry and Molecular Biology, University of Bucharest, Bucharest, Romania; c Department of Physics, University Politehnica of Bucharest, Bucharest, Romania; d Department of Toxicology and Forensic Sciences, Faculty of Medicine, University of Crete, Heraklion, Greece; e Department of Surgery, “Sf. Ioan” Clinical Emergency Hospital, Bucharest, Romania; f Department II, Faculty of Dental Medicine, “Carol Davila” University of Medicine and Pharmacy, Bucharest, Romania; g D. Mendeleev University of Chemical Technology of Russia, Moscow, Russia

**Keywords:** Bacterial cellulose, chitosan, core-shell nanoparticles, biocomposite, dermal fibroblast, wound dressing

## Abstract

Dressing biomaterials play a key role in wound management keeping a moisture medium and protecting against external factors. Natural and synthetic materials could be used as dressings where chitosan and bacterial cellulose is one of the most important solutions. These biopolymers have been used for wound dressing based on their non-toxic, biodegradable, and biocompatible features. In this study, biocomposites based on bacterial cellulose and chitosan membranes tailored with antimicrobial loaded poly(*N*-isopropylacrylamide)/polyvinyl alcohol nanoparticles were prepared. Core-shell polymeric nanoparticles, bacterial cellulose/chitosan membranes, and biocomposites were independently loaded with silver sulfadiazine, a well-known sulfonamide antibacterial agent used in the therapy of mild-to-moderate infections for sensitive organisms. The chemistry, structure, morphology, and size distribution were investigated by Fourier transformed infrared spectroscopy (FTIR-ATR), RAMAN spectroscopy, Scanning electron (SEM) and Transmission electron microscopy (TEM), and Dynamic light scattering (DLS). *In vitro* release behaviors of silver sulfadiazine from polymeric nanoparticles and biocomposites were investigated. The biological investigations revealed good biocompatibility of both the nanoparticles and the biocomposites in terms of human dermal fibroblasts viability and proliferation potential. Finally, the drug-loaded polymeric biomaterials showed promising characteristics, proving their high potential as an alternative support to develop a biocompatible and antibacterial wound dressing.

## 1. Introduction

The last decades brought rapid progress of biomedical polymeric materials field which addresses both natural and synthetic polymers. New approaches try to overcome daily medical challenges, such as drug delivery systems, tissue engineering, or wound dressing. The wounds are generally regarded as a disruption of the skin epithelial cells continuity. The healing process represents a dynamic and complex interaction between cells, growth factors, blood, or extracellular matrix. In some clinical conditions, such as diabetes or extended cancers dermal wounds like ulcers, burns or even surgical cuts frequently do not heal and become a problem that demands dedicated clinical care. Without such an approach these wounds can lead to delayed healing, infections, and many other conditions that may lead to amputation. With this respect, wound dressings evolved in recent years to meet the requirements for modern tissue repair and regeneration. The repairing process requires several steps, such as coagulation, inflammation, proliferation, and maturation. To accomplish these steps and to avoid the microorganisms’ invasion into the wound, the use of an appropriate dressing is crucial (Robson et al., 2001; Eming et al., 2002; Czaja et al., 2007; Dhivya et al., 2015). The selection of proper materials for designing wound dressing is essential for reaching fast healing. Natural polymers, such as microbial derivate polysaccharides (i.e. alginate, dextran, chitosan, bacterial cellulose) (Ahmad et al., 2015; Moscovici, 2015; Fu et al., 2016; Liu et al., 2017; Qi et al., 2021) exhibit promising physical, chemical, and biological properties. These advantages promote them for particularly usage in different biomedical applications like soft tissue engineering, meshes, membranes, or drug delivery (Rinaudo, 2006; Andrade et al., 2010; Bhattarai et al., 2010; Bäckdahl et al., 2011; Almeida et al., 2014; Scherner et al., 2014; Elieh-Ali-Komi & Hamblin, 2016). There are various types of wound dressing systems that enable multiple approaches, such as self-assembling, antibacterial property, injectable, compressive/stretchable, or conductive. All these approaches demonstrated excellent repair activity with respect to classic wound dressing systems. In this regard, natural and natural-derived polymers have gained a central role in designing such wound dressing successful capabilities (Qu et al., 2018; Li et al., 2020; Zhang et al., 2020; Al-Kaabi et al., 2021; Liang et al., 2021; Zhao et al., 2021).

Among these natural polymers, chitosan, and especially bacterial cellulose (BC) proved to be efficient solutions for the treatment of chronic wounds and burns (Czaja et al., 2006; Cui et al., 2011; Jayakumar et al., 2011; Wen et al., 2015; Fürsatz et al., 2018; Portela et al., 2019; Al-Musawi et al., 2020). They can maintain a moist environment at the wound surface, remove the wound excess exudates, prevent infection, or allow water and gases to exchange (Al-Musawi et al., 2020). The chitin and its derivate, chitosan, have several biomedical application requirements, such as biocompatibility, biodegradability, non-toxicity, or hydration (Al-Musawi et al., 2020). Chitosan has numerous advantages for biomedical applications, such as biocompatibility and biodegradability, antimicrobial, antioxidant, and anticancer properties (Singh & Ray, 2000; Zhao et al., 2018; Del Prado-Audelo et al., 2020). Chitosan can provide a non-protein matrix for tissue growth in a 3 D manner and activates macrophages. It was proved that chitosan can accelerate the regeneration of various tissues with wound contraction (Kevin et al., 1999; Del Prado-Audelo et al., 2020) by stimulating inflammatory cells, macrophages, and fibroblasts. Furthermore, chitosan exhibits a hemostatic behavior for blood clotting and reduces pain by blocking nerve ending (Ong et al., 2008; Radwan-Pragłowska et al., 2019). The hemostatic properties could be attributed to the positive charge of chitosan, whereas the red blood cells are negatively charged. Therefore, these cells could interact with chitosan macromolecules by electrostatic interactions. Finally, it is worth mentioning that chitosan can be easily processed into hydrogels, membranes, fibers or sponges making it a very attractive biomaterial for various applications in the biomedical field (Portero et al., 2007; Nagahama et al., 2008; Jayakumar et al., 2009; Peter et al., 2010; Hasan et al., 2020).

Bacterial cellulose has been obtained from an exopolysaccharide by *Komagataeibacter xylinus* bacteria for different applications, but especially for the medical field. It is obtained in pure form as compared to plant cellulose which contains lignin, pectin, and hemicellulose. It is a unique polysaccharide among natural microbial polymers due to its special structural features and physico-mechanical properties. The unique structure based on a fibril network has a special 3 D arrangement capable to produce multilayers of bacterial cellulose with high surface area and high porosity (Ul-Islam et al., 2012; Weyell et al., 2019; Muthu & Rathinamoorthy, 2021). Based on its special mechanical (tensile) and chemical properties, bacterial cellulose can provide a high wound dressing protection by immediately wound closure. Another important characteristic of bacterial cellulose is related to its biocompatibility, permeability and maintaining wound hydrated and safe from infection. Besides skin problems as an acute or chronic wound, skin burns are considered highly complex injuries with intense damaging of skin tissue. In 2016, American Burn Association reported almost 500,000 burn injuries with roughly 40,000 hospitalizations. In the case of burns, the literature data on bacterial cellulose used as wound dressing provided successfully healing results by faster re-epithelization of wound (Balasubramani et al., 2001; Malcolm Brown & Saxena, 2007; Sulaeva et al., 2015; Weyell et al., 2019; Zheng et al., 2020). Wound dressings based on bacterial cellulose biomaterials are currently used or applied in infected and diabetic wounds, burns, cancer lesions, skin trauma, revealing exceptional therapeutic effects on promoting wound healing (Kucińska-Lipka et al., 2015; Sulaeva et al., 2015; Portela et al., 2019; Ahmed et al., 2020; He et al., 2020; Zheng et al., 2020; Asanarong et al., 2021).

In this context, the paper aims to develop novel wound dressing biocomposites able to substitute the classic dressings by offering complex therapy management through the synergistic effect of the bacterial cellulose/chitosan membrane and delivery of an active compound at the wound site. The membrane support as a wound dressing can exhibit adequately prolonged close contact between dressing and skin area around the wound. The proposed membrane support exhibits adequately prolonged close contact between the dressing and the injured skin area while the entrapped core-shell nanoparticles proposed in this model provide a controlled release of active principles for antibacterial protection and acceleration of wound repair.

## 2. Materials and methods

### 2.1. Materials

Low molecular weight chitosan (50,000–190,000 Da) with a 75% percent of deacetylation, anhydrous calcium chloride, sodium hydroxide, polyvinyl alcohol (PVA) with 88% hydrolysis degree (88,000 Da), *N*-isopropylacrylamide (NIPAM), methyl oleate (MO), and potassium persulfate initiator were purchased from Sigma–Aldrich, 3050 Spruce Street, St. Louis, MO 63103, USA. Bacterial cellulose (BC) was provided by National Chemical-Pharmaceutical for Research and Development Institute, Bucharest, Romania. Silver sulfadiazine drug was purchased from ACROS Organics, New Jersey, USA.

### 2.2. Synthesis of poly(N-isopropylacrylamide) nanoparticles

The synthesis of poly(*N*-isopropylacrylamide)/polyvinyl alcohol/methyl oleate (PNIPAM/PVA/MO) nanoparticles is based on a radical polymerization mechanism of the corresponding monomer and polyvinyl alcohol (PVA) and methyl oleate (MO). Briefly, aqueous solutions of polyvinyl alcohol (0.5 and 5 wt. % concentration) were prepared. In the meantime, aqueous solutions of *N*-isopropylacrylamide monomer (NIPAM) with 0.5 and 5 wt. % concentration was prepared, and the potassium persulfate initiator (KPS) was dissolved accordingly. Solutions of NIPAM/PVA 5 wt. % and NIPAM/PVA 0.5 wt. % were prepared by stirring for 2–3 h. After obtaining NIPAM/PVA 0.5 and 5% solutions, the mixtures were divided into four parts and methyl oleate (MO) was added in ratios 0.125, 0.25, 0.5, and 1 v/v within the mixture (Table 1). *N*-isopropylacrylamide/polyvinyl alcohol/methyl oleate (NIPAM/PVA/MO) solutions were subjected to vigorous stirring for 12 h to prepare the nanoparticles suspension. The NIPAM/PVA/MO nanoparticles suspension was heated at 60 °C for 4–5 h for NIPAM grafting and final stabilization of nanoparticles suspension. The final systems were obtained as core-shell nanocapsules with a methyl oleate core, PVA interfacial stabilizer, and PNIPAM as a polymeric shell. The layered core-shell structuration is beneficial to get nanocarriers with surface entrapped drugs for higher release efficiency (Li et al., 2009).

**Table 1.** Recipes for biocomposites’ preparation.

Sample no.	BC/chitosan ratio w/w	(BC/chitosan)/Nanoparticles ratio w/w	Nanoparticles: NIPAM/PVA conc (%)	Methyl oleate (%)
S7	15/85	85/15	0.5	0.125
S8	15/85	85/15	5	0.125
S9	15/85	70/30	0.5	0.125
S10	15/85	70/30	5	0.125

### 2.3. Drug loading of PNIPAM/PVA/MO nanoparticles

The prepared PNIPAM/PVA/MO nanoparticles with various polymer and methyl oleate concentrations were loaded with silver sulfadiazine, an antimicrobial agent for skin infections (Hussain & Ferguson, 2006; Munteanu et al., 2016). The silver sulfadiazine drug was added into PNIPAM/PVA/MO nanoparticles suspension under vigorous stirring for 12 h. Afterward, the drug-loaded PNIPAM/PVA/MO nanoparticles suspension was dried at 60 °C for 24 h for water removal and nanoparticles recovery.

### 2.4. Preparation of bacterial cellulose/chitosan membranes

**Figure 1.**
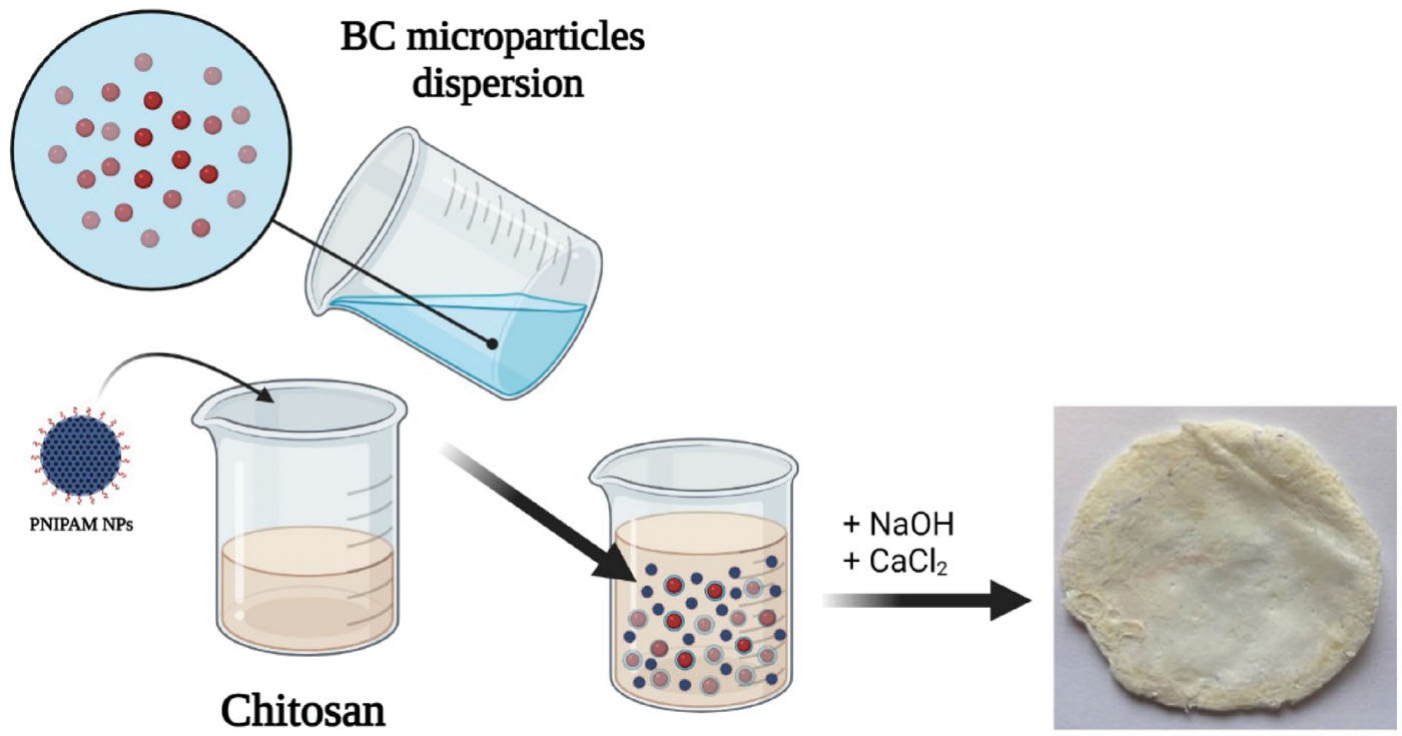
Steps in the preparation of BC/chitosan biocomposites tailored with drug-loaded PNIPAM nanoparticles.

Preparation of polymeric membranes starts from chitosan aqueous solution and bacterial cellulose aqueous dispersion (Figure 1). Briefly, a low molecular weight chitosan solution (7 wt. %) was prepared in an aqueous acid medium with a ratio of 1/10 (v/v) of hydrochloric acid/distilled water in 4 h at 60 °C. The acid medium is more suitable for obtaining chitosan solution with a transparent aspect and a higher concentration. The bacterial cellulose was prepared as an aqueous dispersion of BC microparticles by micro-fluidization technique, where a high-pressure homogenization process was applied with several recirculation steps. BC suspension was subjected to heating-cooling cycles (3–4 cycles: −20/60 °C) for high concentration (4%) and water evaporation. The viscous solution of chitosan and BC dispersion in ratios of 85/15 w/w were mixed for 1 h. The resulted mixture was cast onto glass flasks to obtain membranes. In parallel, 5% sodium hydroxide and 10% calcium chloride solutions were prepared (ratio 2:1 to the membrane mass (v/w). The sodium hydroxide solution was dropwise added onto BC/chitosan mixture to induce chitosan cross-linking and BC basic treatment for 10 min. Afterward, the sodium hydroxide solution was removed, and the calcium chloride solution was dropwise added for precipitation of previously treated BC for 10 min. The obtained BC/chitosan membranes were thoroughly washed with water to remove the remaining salts.

### 2.5. Preparation of biocomposites based on BC/chitosan membranes tailored with drug-loaded PNIPAM/PVA/MO nanoparticles

The drug entrapped biocomposites were obtained by the previously described method. The suspension of drug-loaded PNIPAM/PVA/MO nanoparticles was added during the stirring step of viscous chitosan solution and BC particles suspension (Figure 1). This time, all the three parts, chitosan solution, BC dispersion, and drug-loaded nanoparticles were mixed, followed by casting on glass plates. Finally, they were subjected to treatment in sodium hydroxide and calcium chloride solution for 10 min (Figure 1). The biocomposites were thoroughly washed with distilled water to remove the remaining salts and then subjected to characterization by various methods.

### 2.6. Characterization methods

#### 2.6.1. FTIR and RAMAN

FTIR spectra of native and treated bacterial cellulose (BC), native and treated chitosan, and BC/chitosan membranes were recorded on a Bruker Vertex 70 FT-IR spectrophotometer with attenuated total reflectance (ATR) accessory with 32 scans and 4 cm^−1^ resolutions in the mid-IR region. Raman spectra were recorded on a DXR Raman microscope, from Thermo Fisher Scientific. The excitation laser wavelength was 532 nm using a laser power of 14 mW. The Raman spectra were collected in the range of 100–3200 cm^−1^ with a relevant display in the range 800–1750 cm^−1^.

#### 2.6.2. Morphological characterization by scanning electron microscopy (SEM) and transmission electron microscopy (TEM)

The size and morphology of the nanoparticles together with membrane morphological characteristics were investigated by Scanning Electron Microscopy (SEM) using a Quanta Inspect F50, with a field emission gun (FEG) having 1.2 nm resolution and an Energy Dispersive X-ray Spectrometer (EDXS) having 133 eV resolution at MnKα. Morphology, geometrical evaluation (size and shape) of nanostructural characteristics of the core-shell constructs were investigated by high-resolution transmission electron microscopy (HR-TEM) and selected area electron diffraction (SAED) using a TECNAI F30 G2 S-TWIN microscope operated at 300 kV with Energy Dispersive X-ray Analysis (EDAX) facility.

#### 2.6.3. XRD analysis

X-ray diffraction (XRD) spectra were registered on a Panalytical X’PERT MPD X-ray Diffractometer, in the range, 2*θ* = 10–80. An X-ray beam characteristic of Cu Kα radiation was used (*λ* = 1.5418 Å).

#### 2.6.4. Swelling measurements

The swelling behavior of the biocomposites was evaluated in saline solution at 37 °C. The weight changes of the samples were recorded at regular time intervals during swelling. The swelling degree of the hydrogels was determined according to the following equation:

(1) $$SD=\frac{W_{t}-W_{o}}{W_{0}}\cdot 100\%$$

where *W_t_* and *W_0_* denote the weight of the wet hydrogel at a predetermined time and the weight of the dry sample, respectively. The equilibrium swelling degrees (ESD) were measured until the weight of the swollen hydrogels was constant. At least three swelling measurements were performed for each sample and the mean values were reported. Furthermore, gel fraction experiments were performed by leaching tests.

The gel fraction (GF) of the samples was determined according to the following equation (Wong et al., 2015):

(2) $$GF=\frac{W_{f}}{W_{0}}\cdot 100\%$$

where *W_f_* denotes the weight of the dried sample after water extraction.

#### 2.6.5. Evaluation of the rheological properties

Rheological tests have been performed with a rotational rheometer Kinexus Pro, Malvern Instruments, and a temperature control unit. In oscillating mode, a parallel plate and a geometric measuring system have been used, and the gap has been set according to the force value. The tests were performed on samples of 20 mm diameter with parallel plate geometry in a frequency range of 1–20 Hz. Shear viscosity measurements were performed at a fixed shear rate of 0.1 s^−1^.

#### 2.6.6. Evaluation of the static mechanical properties by tensile tests

Mechanical tensile tests were performed on an Instron 2519-107 Universal testing machine equipped with a 5 kN load cell. The measurements were done at room temperature on dumbbell specimens with a crosshead rate of 10 mm/min. Data were collected for at least three specimens for each sample with 0.5% accuracy of force measurement and position accuracy of 0.001 mm. Specimens were prepared according to ISO 527-2012 (overall length 75 mm, gauge length 25 mm, width 5 mm, and thickness 2 mm) (Hervy et al., 2017).

#### 2.6.7. Dynamic light scattering and zeta potential

The size distribution and zeta potential were investigated by Dynamic Light Scattering (DLS) using a Zetasizer Malvern DLS device. Data were collected for PNIPAM nanoparticles obtained from 0.5 and 5 wt. % polymer concentration.

#### 2.6.8. Drug encapsulation and in vitro release behavior

The silver sulfadiazine encapsulation within both nanoparticles and BC/chitosan membranes followed two pathways: in the case of nanoparticles, the drug was dissolved into Phosphate buffer saline (potassium phosphate/sodium hydroxide) (PBS) solution (pH 7.45). The drug solution concentration was: 0.1 mg/mL for nanoparticles from 5% polymer concentration and 0.05 mg/mL for nanoparticles from 0.5% polymer concentration. The dried nanoparticles were added to the drug solution (0.1 g of nanoparticles/mL of drug solution). The resulted nanoparticle suspension was stirred for 24 h. Finally, the nanoparticles were separated by filtration. In the second case, the dried bacterial cellulose/chitosan membrane was immersed into drug solution and the encapsulation took place by swelling. For both cases, the encapsulation evaluation was done by UV–VIS analysis of the drug that remained in the solution. *In vitro* release behaviors of silver sulfadiazine from PNIPAM/PVA/MO nanoparticles and biocomposites were evaluated in time. Briefly, the dried loaded PNIPAM/PVA/MO nanoparticles and subsequently membrane biocomposites were entrapped in a cellulose membrane, immersed in 50 mL of PBS (0.01 M, pH 5 to reproduce the skin pH), and incubated in a precision water bath (orbital mixer Benchmark Scientific) at 400 rpm and 37 °C. Aliquots (5 mL) containing a mixture of PBS and released drug were collected at defined time points and the release medium was refreshed with the addition of an equal amount of fresh PBS after each withdrawal to maintain the total volume of the sample constant. The silver sulfadiazine release profiles from nanoparticles and biocomposites were evaluated by UV-VIS spectroscopy.

### 2.7. Cell culture model

Considering the final application of the novel biocomposites in the field of wound healing, human dermal fibroblasts (CCD-1070Sk, ATCC^®^ CRL-2091) were used as *in vitro* cell culture model to screen the cytotoxicity potential of the developed nanoparticles and to evaluate the biocompatibility of the proposed biomaterials. In this view, CCD-1070Sk human dermal fibroblasts were grown in Minimum Essential Medium (MEM), supplemented with 1% (v/v) penicillin/streptomycin (10,000 U/mL penicillin and 10 mg/mL streptomycin) and 10% (v/v) fetal bovine serum (FBS) in a humidified atmosphere of 5% CO_2_, at 37 °C. The culture medium was refreshed every two days and cells were split 1:3 weekly, by trypsin/EDTA treatment.

### 2.8. Core-shell polymeric nanoparticles cytotoxicity screening

The cytotoxic potential of the poly(*N*-isopropylacrylamide)/polyvinyl alcohol nanoparticles was investigated after 24 and 48 h of treatment with pristine PNIPAM/PVA/MO. In this view, 10^4^ cells/cm^2^ were seeded in well plates and allowed to adhere to the substrate for 24 h. The next day, the culture media were exchanged with the following PNIPAM/PVA/MO dilutions prepared in complete culture medium: 20, 15, 10, 7.5, and 5 mg/ml. These treatments were applied 24 h post-seeding and maintained for 24 and 48 h.

#### 2.8.1. MTT assay

To evaluate the amount of the metabolically active cells as a measure of cell viability under the treatment with various concentrations of PNIPAM/PVA/MO, a quantitative spectrophotometric MTT assay was employed. Briefly, after 24 and 48 h of treatment, cell culture media were harvested and the CCD-1070Sk monolayers were washed with a serum-free culture medium. The samples were then incubated for 4 h at 37 °C in 1 mg/ml MTT solution. After this step, the formazan crystals produced by the metabolically active cells were solubilized in DMSO. The absorbance of the resulting solutions was determined at 550 nm by using FlexStation III multimode microplate reader (Molecular Devices). An untreated control was prepared following the same procedure and used as a reference.

#### 2.8.2. Live and dead fluorescence microscopy assay

This test was employed as an additional viability investigation to evaluate the ratio between the living and the dead cells. In this view, after 24 and 48 h of treatment, cell culture media were harvested and the CCD-1070Sk monolayers were washed with a serum-free culture medium. A mixed solution of calceinAM and ethidium bromide was prepared according to the manufacturer’s recommendations (LIVE/DEAD Assay, ThermoFischer Scientific, Waltham, MA, USA). All the samples, including the untreated reference, were incubated for 30 min at room temperature and darkness with this staining solution. In the end, the monolayers were investigated using an Olympus IX73 inverted fluorescence microscope and CellSense imaging software for image capturing.

#### 2.8.3. Lactate dehydrogenase activity (LDH assay)

The cytotoxic potential of the PNIPAM/PVA/MO on CCD-1070Sk human dermal fibroblasts was investigated by spectrophotometric evaluation of Lactate Dehydrogenase (LDH) activity in the culture media after 24 and 48 h of treatment. Briefly, 500 µl of culture media were harvested at the above-mentioned time points and mixed with the components of the *In Vitro* Toxicology Assay Kit (TOX-7 kit, Sigma Aldrich, Saint Louis, MO, USA) according to the manufacturer’s instructions. After 20 min of incubation in darkness at room temperature, the absorbance of the samples was determined at 490 nm using FlexStation III (Molecular Devices) microplate reader.

### 2.9. Biocompatibility assessment of biocomposites

A preliminary biocompatibility investigation of the biocomposites was conducted by performing the MTT assay. In this view, human dermal fibroblast cells from CCD-1070Sk cell line were seeded in direct contact with the biomaterials at an initial density of 10^6^ cells/sample.

#### 2.9.1. MTT assay

After 24 h and 5 days of culture, cell viability was investigated. In this view, cell culture media were harvested and the monolayers from the membranes’ surfaces were washed with a serum-free culture medium. The samples were then incubated for 6 h at 37 °C in 1 mg/ml MTT solution. The formazan crystals produced by the metabolically active cells were solubilized in DMSO and the absorbance of the resulting solutions was determined at 550 nm by using FlexStation III multimode microplate reader (Molecular Devices).

### 2.10. Statistical analysis

All the *in vitro* experiments were performed in triplicate (*n* = 3) and results were analyzed using one-way ANOVA and Bonferroni test. All the results were presented as mean value ± standard deviation (SD) using GraphPad Prism Software 6.0 (GraphPad Software Inc., La Jolla, CA, USA).

## 3. Results and discussions

### 3.1. FTIR and RAMAN analysis

#### 3.1.1. FTIR

**Figure 2.**
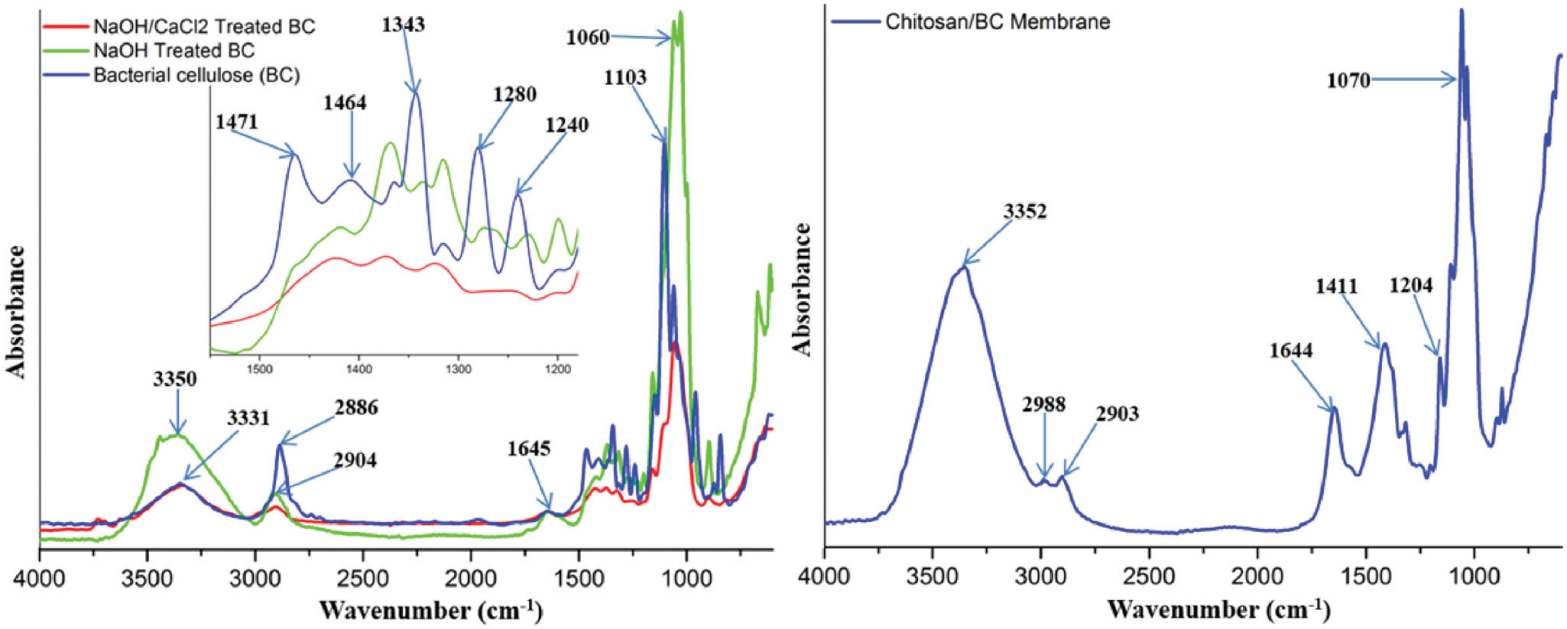
FTIR spectra of treated/untreated bacterial cellulose (individual component) (left); and final BC/chitosan membrane (right).

**Figure 3.**
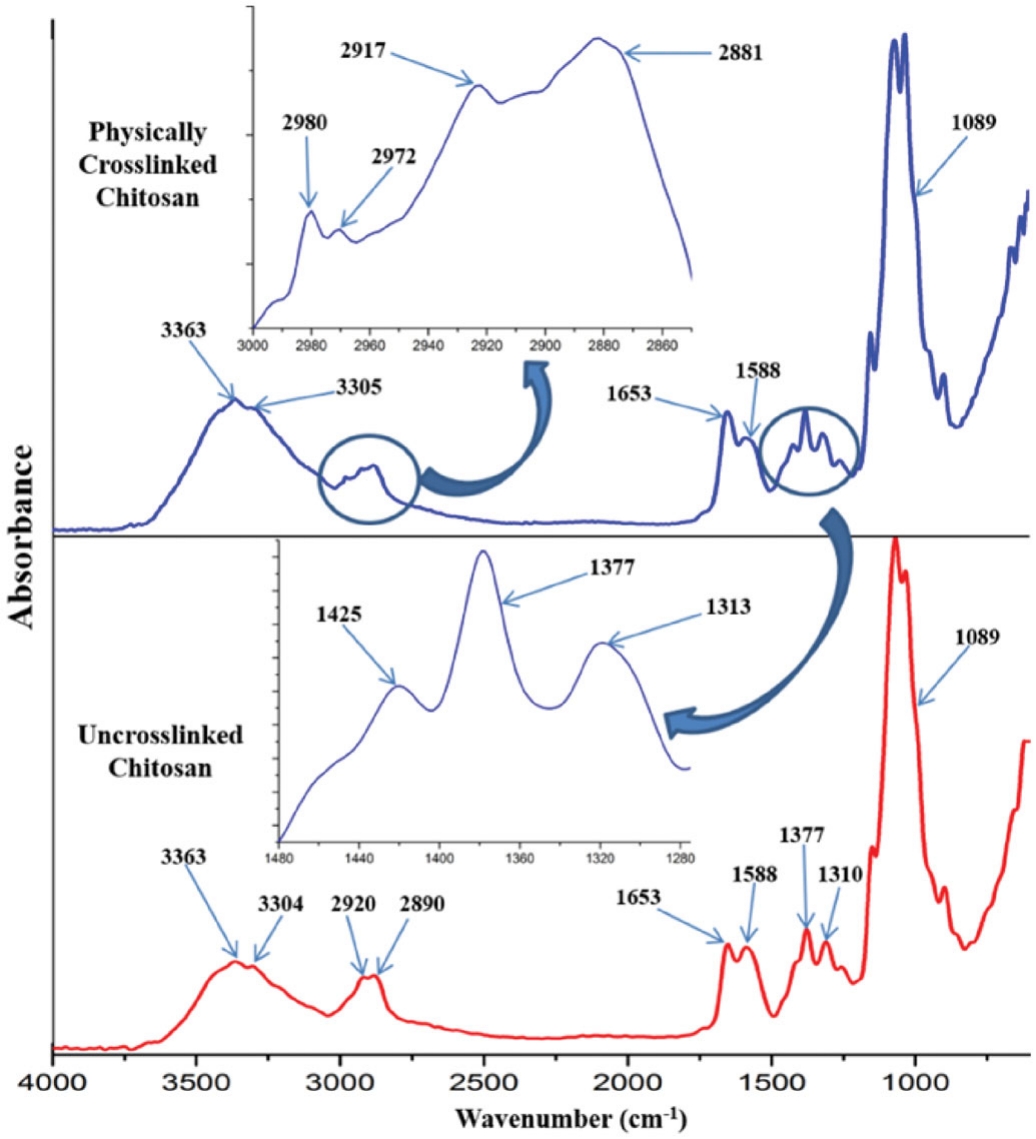
FTIR spectra of uncrosslinked and physically crosslinked chitosan (individual component).

The physico-chemical characterization of BC/chitosan membranes assumes characterization of each polymer and its interaction with sodium hydroxide and calcium chloride agents. FTIR spectrum of native bacterial cellulose (Figure 2) revealed the following peaks: an intense wide peak at 3331 cm^−1^ specific for O–H stretching from hydroxyl bacterial cellulose groups and an intense peak at 2886 cm^−1^ attributed to symmetric stretching of C–H bond from methylene and methine groups. The maximum at 1645 cm^−1^ corresponds to hydroxyl group bending vibration including absorbed water molecules. The peaks at 1471, 1464, and 1342 cm^−1^ are attributed to methyl and methine bending vibration from the backbone. The peaks at 1280 and 1240 cm^−1^ were attributed to the stretching vibration of C–O bonds. The intense peak at 1103 cm^−1^ is attributed to C–O–C ether bonds from piranozic cycles backbone and between piranozic cycles (Auta et al., 2017; Hospodarova et al., 2018). After treatment with sodium hydroxide, an increase of the peak intensity was observed at 3350 and 1645 cm^−1^ specific for stretching and respectively, bending vibrations from hydroxyl groups. Probably the basic medium led to the decrease of physical interactions between hydroxyl groups from bacterial cellulose structure. The treatment with calcium chloride led to the decreasing intensity of peaks at 3350 and 1645 cm^−1^ specific for stretching and respectively, bending vibrations from hydroxyl groups. Thus, this result can be explained by the participation of these groups in new ionic interactions between calcium cations Ca^2+^ and treated negatively charged alcoholate ions –O^-^. This led to the formation of stabilized/precipitated bacterial cellulose. These ionic interactions are important for the integration of BC to the membrane structure resulting in an interpenetrating network. Without calcium stabilization, the bacterial cellulose could exude the membrane in aqueous environments leading to membrane de-structuring. The FTIR spectrum of chitosan (Figure 3) revealed the following specific maxima: peaks at 3363 and 3304 cm^−1^ attributed to O–H stretching from hydroxyl groups and respectively stretching vibration of N–H bonds from amino groups. The peaks at 2920 and 2890 cm^−1^ were attributed to the asymmetric stretching vibration of C–H from methylene groups, respectively to the symmetric stretching vibration of C–H from methyl groups. The peak at 1653 cm^−1^ was attributed to the stretching vibration of C = O from carbonyl in acetylated groups. The peaks at 1588 and 1377 cm^−1^ were assigned to bending deformation of N–H bond from amino groups, respectively to bending deformation of C–H from methyl and methylene. The peak at 1310 cm^−1^ was attributed to stretching vibration of C–N bonds. The intense peak at 1089 cm^−1^ was attributed to stretching vibration of C–O and ether C–O–C bonds (Fernandes Queiroz et al., 2014; Jóźwiak et al., 2017). The treatment with sodium hydroxide followed a physical crosslinking of chitosan specific to the obtaining of a stabile hydrogel. Various results are shown in literature for the preparation of physical crosslinked chitosan hydrogels with different mechanisms (Cerchiara et al., 2002; Hennink & van Nostrum, 2002; Kumirska et al., 2010; Ladet et al., 2011; Ostrowska-Czubenko et al., 2013; Nilsen-Nygaard et al., 2015). FTIR spectra highlighted three new peaks in the regions 2980, 2972, and 1425 cm^−1^ which can be attributed to the asymmetric stretching vibration of C–H from methyl groups, respectively for bending vibration of C–H from methyl groups. The single available methyl groups are present in the acetate groups. This result could suggest that acetate groups contributed to the generation of a new physical cross-linked network with hydrogen bonding. The physical crosslinked network mechanism may be explained by a dual intermolecular acetate interaction shown in Figure 2. The participation of acetate groups in hydrogen bonding led to new contributions in FTIR spectra of contained methyl groups besides peaks at 2881 and 1377 cm^−1^. Furthermore, the peak at 1653 cm^−1^ specific for C = O stretching, has an increased intensity due to the contribution to hydrogen bonding. The increasing intensity of peaks at 3363 and 3305 cm^−1^ may suggest the implication of hydroxyl and amino groups in hydrogen bonding. The basic medium (pH 12) generated by sodium hydroxide neutralized the –NH_3_^+^ species needed for chitosan dissolution. The –NH_3_^+^ species neutralization supported by the high polymer concentration (7%) and deacetylation degree (75%) ensured the optimal conditions for the generation of the novel physically crosslinked network. Thus, there were developed structural and dimensional stable membranes in specific basic medium. The BC/chitosan membrane spectrum (Figure 2) revealed a wide intense specific peak at 3352 cm^−1^ attributed to stretching vibration of O–H and N–H bonds, two peaks at 2988 and 2903 cm^−1^ attributed to the asymmetric stretching vibration of C–H bond from methyl groups and respectively, from methylene groups. The peak at 1644 cm^−1^ is specific for stretching vibration of C = O from carbonyl in acetylated groups. The next peaks at 1411 and 1070 cm^−1^ were assigned to bending vibration from methyl and methylene groups, respectively to stretching vibration of C–O and ether C–O–C groups from piranozic backbone.

#### 3.1.2. RAMAN

**Figure 4.**
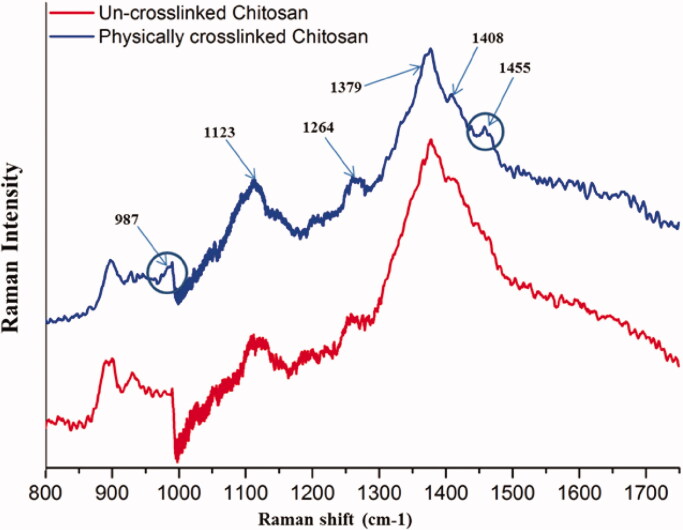
RAMAN spectra of uncrosslinked and physical crosslinked chitosan.

The uncrosslinked and physically crosslinked chitosan were further characterized by RAMAN to highlight the interactions leading to the 3 D physical network. The RAMAN spectra (Figure 4) of chitosan revealed the main peaks: at 1123 cm^−1^ attributed to stretching C–O–C bonds, stretching C–O bonds from piranozic backbone, and stretching C–OH. The next peak at 1264 cm^−1^ is assigned to C–N stretching, 1379 cm^−1^ is assigned to CH_2_ and CH bending, and 1408 cm^−1^ is assigned to CH_3_ bending. The new peaks at 987 and 1455 cm^−1^ were attributed to out-of-plane and, respectively bending vibration of CH_3_, CH_2_, and CH groups (Zając et al., 2015).

### 3.2. X-Ray diffraction (XRD)

**Figure 5.**
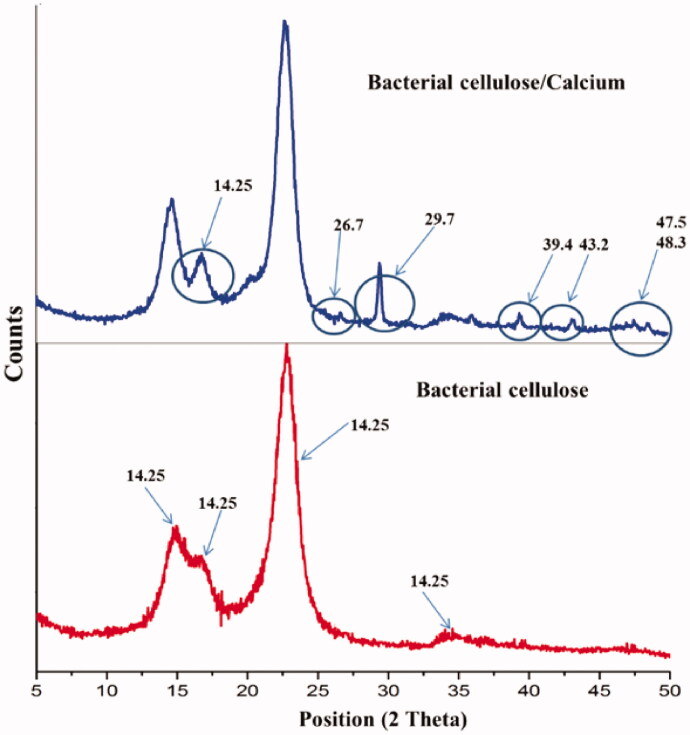
XRD diffractograms of bacterial cellulose and calcium treated bacterial cellulose and treated bacterial cellulose (sodium hydroxide and calcium chloride solutions).

The interaction of bacterial cellulose (BC) with calcium cations was further investigated by XRD. The diffractograms of bacterial cellulose and calcium-treated bacterial cellulose are shown in Figure 5. They revealed the main peaks for BC in accordance with literature data (Cheng et al., 2009; Zaharia et al., 2014; Tsouko et al., 2015), but specific for chopped BC microparticles and not as a classical membrane. The main diffraction peaks were seen at 2 theta of 15.56° and a shoulder at 16.78° assigned to diffraction plane (101), 22.9° assigned to diffraction plane (002), and two low-intensity peaks at 34.3 and 35.9°. For calcium treated BC new peaks were observed which can be attributed to the interactions between BC and calcium cation and newly formed crystalline structure. Considering the BC-calcium interactions as ionic interactions of Ca^2+^ and treated negatively charged alcoholate ions –O^−^, the new peaks were compared with calcium oxide (CaO) crystalline structure. The new peaks at 2 theta of 26.7, 29.7, 39.4, 43.2, 47.5, and 48.3° completed the file of calcium oxide crystalline structure. Furthermore, the peak at 14.55° was split into two independent peaks probably due to a more organized crystalline structure of native BC with calcium contribution. XRD results revealed major interactions of BC with calcium cations leading to a stabilized BC network which can be considered as a pseudo-crosslinked structure with the association of microparticles into a unitary structure. The pseudo-crosslinking of BC microparticles into a unitary structure had a major role in the design of (BC)/chitosan membranes as interpenetrated network (IPN).

### 3.3. Evaluation of the static tensile properties of chitosan membrane

**Figure 6.**
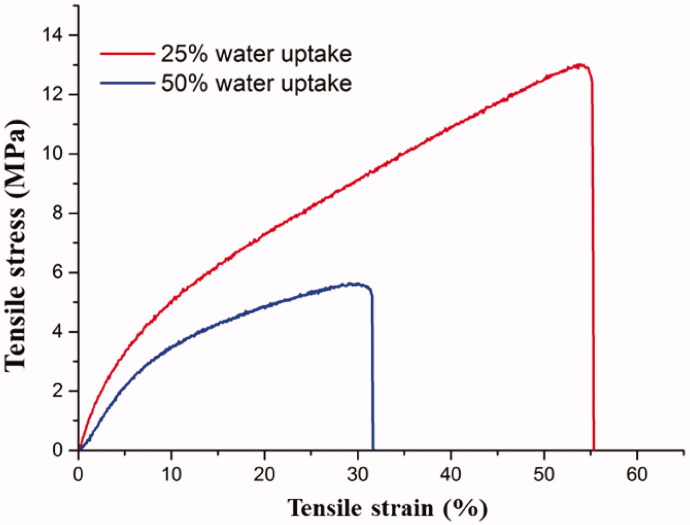
Stress-strain curves of physically crosslinked chitosan samples with different water uptake (25 and 50%).

The evaluation of the mechanical properties of physically crosslinked chitosan membrane is an interesting approach for a further understanding of the 3 D network. There are too many tensile assays in the literature on physically crosslinked chitosan (Jin et al., 2004), but the performed tensile test served to compare the results on the novel 3 D network. The tensile results (Figure 6) revealed two tests on partial swollen chitosan samples (25 and 50%). The partial swollen sample with 25% water uptake exhibited a tensile strength of about 12.9 MPa and the sample with 50% water uptake exhibited a tensile strength of about 5.1 MPa. The increase of water uptake led to the decrease of tensile strength and elongation probably because the water molecules act by collapsing the physical bridges with decreasing the network density. Moreover, the tensile strength values are doubled as compared to literature data for physical crosslinking (Portero et al., 2007) and in the same range with chemical crosslinking (Aishah Binti Mohd Isa & Mohamed, 2015; Miles et al., 2016; Gadgey & Sharma, 2018), resulting in a more resistant material. Thus, the tensile results sustained the development of a novel stronger physical network of chitosan.

### 3.4. Swelling measurements on chitosan membrane and gel fraction analysis

**Figure 7.**
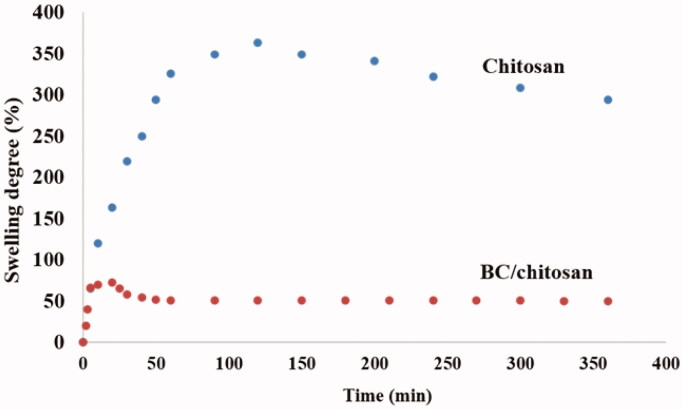
Swelling behavior of physically crosslinked chitosan and BC/chitosan support.

The physically crosslinked chitosan membrane was subjected to swelling tests to investigate the interaction of the physical network with water molecules. The swelling results (Figure 7) showed an unusual behavior which could be explained as follows. The first approach was the high swelling rate for the first 10 min with doubling the sample mass. This behavior can be explained by the hydrophilic nature of glucose-amine rings, chain flexibility, and probably the lack of an ordered arrangement of macromolecular chains. The treatment of chitosan with sodium hydroxide led to very fast physical interactions like chain freezing in a steady position without any possibility for chain rearrangement. The maximum swelling degree value was about 360%. Fast entrapping of water molecules is followed by swelling stability with a maximum degree after 120 min. The second unusual behavior was the slow decrease of swelling degree, a rarely encountered behavior for hydrogels in literature (Nandi & Winter, 2005; Gupta & Shivakumar, 2012). The equilibrium water content was reached at around 280% swelling degree The explanation for the swelling degree decrease could be related to the rearrangement of the frozen chains with a further post-crosslinking. The post-crosslinking step led to the increase of network density with more physical bridges and probably higher mechanical properties which assured an expulsion of water molecules from the network system. The physical crosslinking of chitosan in these conditions can be performed in several steps with a direct influence on the mechanical properties. Furthermore, the mass loss is sustained by the un-crosslinked chain loss. The gel fraction was established at around 56%. The physically crosslinked BC/chitosan membrane revealed a similar behavior, but at far different values. The maximum swelling degree value was rapidly reached (65% in 10 min). The second swelling stage was represented by a swelling degree decrease (40 min) while the last stage was seen at equilibrium (53%). The mass loss was also sustained by the un-crosslinked chain loss. The gel fraction was established at around 69%. The high difference of the two maximum swelling degrees can be explained by the BC-calcium layer formed at the membrane surface which opposed the water entrapment. Furthermore, the BC-calcium interactions can significantly contribute to the membrane network besides the physically crosslinked chitosan. This approach will be the subject of future research.

### 3.5. Evaluation of the rheological properties of chitosan and BC/chitosan membranes

**Figure 8.**
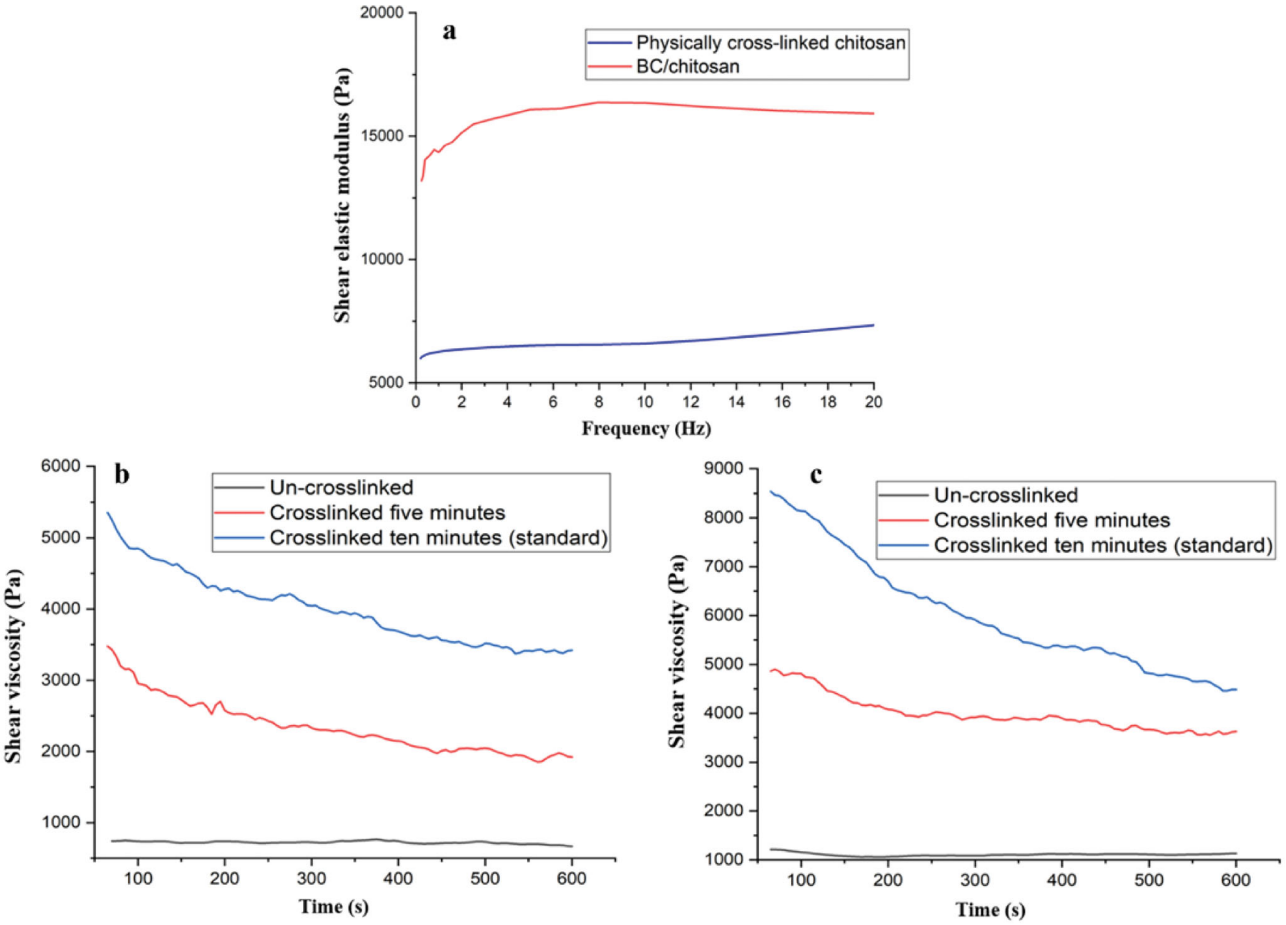
Rheological behavior of physically crosslinked chitosan and BC/chitosan membranes (a); shear viscosity measurements for BC/chitosan (b) and chitosan (c).

The investigation of rheological behavior was performed on swollen samples at swelling equilibrium. The stress was optimized to maintain a linear viscoelastic domain with frequency dependence. Figure 8(a) shows the variation of the elastic modulus of chitosan hydrogel with frequency for dimensional and mechanical stable samples. The elastic modulus of chitosan hydrogel revealed values (5500–6000 Pa) far higher as compared to literature data for physically crosslinked chitosan which are around 100 Pa (Montembault et al., 2005). Furthermore, variation of elastic modulus with frequency revealed a behavior specific for normal chemically crosslinked chitosan with values in the same range or even higher (Weng et al., 2007; Kempe et al., 2008; Ryu et al., 2011; Derkach et al., 2017; Iglesias et al., 2018). BC/chitosan revealed a higher elastic modulus with respect to chitosan hydrogel probably due to the higher network density. This aspect is also sustained by the gel fraction measurements and dual contributions of crosslinked chitosan and BC-calcium interactions. Furthermore, a lower water content for the BC/chitosan membrane can be a reason for the higher network density. To reveal the rheological differences between un-crosslinked and crosslinked samples, several shear viscosity measurements were performed (Figures 8(b,c)). Thus, un-crosslinked and the crosslinked samples for 5 and 10 min were subjected to this analysis. The chitosan hydrogels were crosslinked in sodium hydroxide for 5 and 10 min as standard time. The BC/chitosan membranes were crosslinked in sodium hydroxide and calcium chloride for 5 and 10 min as standard time. The un-crosslinked samples revealed the lowest shear viscosity values. This approach can be explained by the network development during crosslinking process. There are differences between 5 and 10 min revealing that crosslinking process is still ongoing. The differences between the shear viscosity values of chitosan hydrogel and BC/chitosan membrane are given by the BC presence which reduces the chitosan normal viscosity. This fact can be seen very well when comparing with un-crosslinked samples (chitosan *vs.* BC/chitosan). The physical crosslinking process could be an ideal approach for future similar systems based on chitosan to express the desired mechanical properties.

### 3.6. Morphological characterization of PNIPAM/PVA/MO nanoparticles by SEM

**Figure 9.**
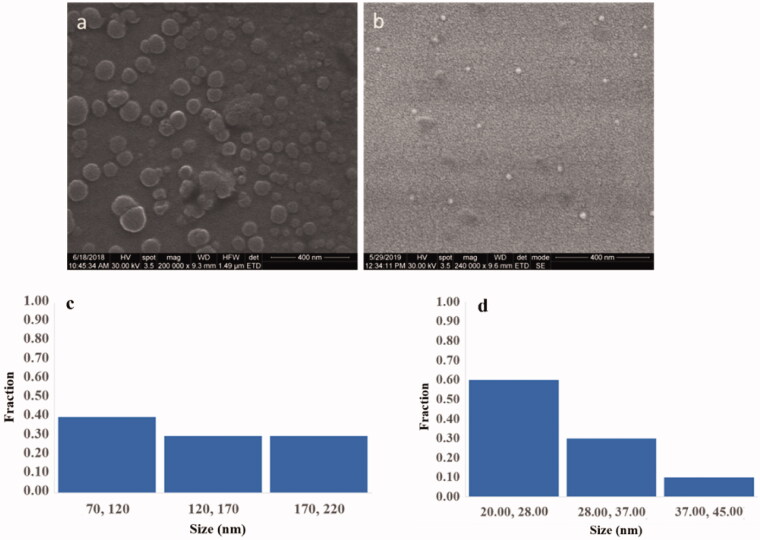
SEM microphotographs of nanoparticles from 0.5% (a) and 5% (b) PNIPAM/PVA concentration with methyl oleate 0.125% concentration; Histogram of size distributions of nanoparticles from 0.5% (c) and 5% (d).

High-resolution SEM images (Figures 9(a,b)) reveal the dimensional information and differences between nanoparticles obtained in different conditions. The nanoparticles obtained from 0.5% NIPAM and PVA solutions and 0.125% MO were in the range of 50–150 nm (Figure 9(c)) with a narrow distribution having a spherical shape and clean surface without any PVA excess traces. One may assume that PVA does not alone contribute to shell structuring, but together with PNIPAM. PVA is known to remain entrapped when used alone as a surface stabilizer (Radu et al., 2019). This result can suggest that PVA polymer acts as a stabilizer and interposes close to the methyl oleate core. The nanoparticles obtained from 5% NIPAM and PVA solutions and 0.125% MO had also a narrow distribution being <50 nm (Figure 9(d)) with a round shape and without PVA excess traces.

#### 3.6.1. Morphological characterization of PNIPAM/PVA/MO nanoparticles by TEM

**Figure 10.**
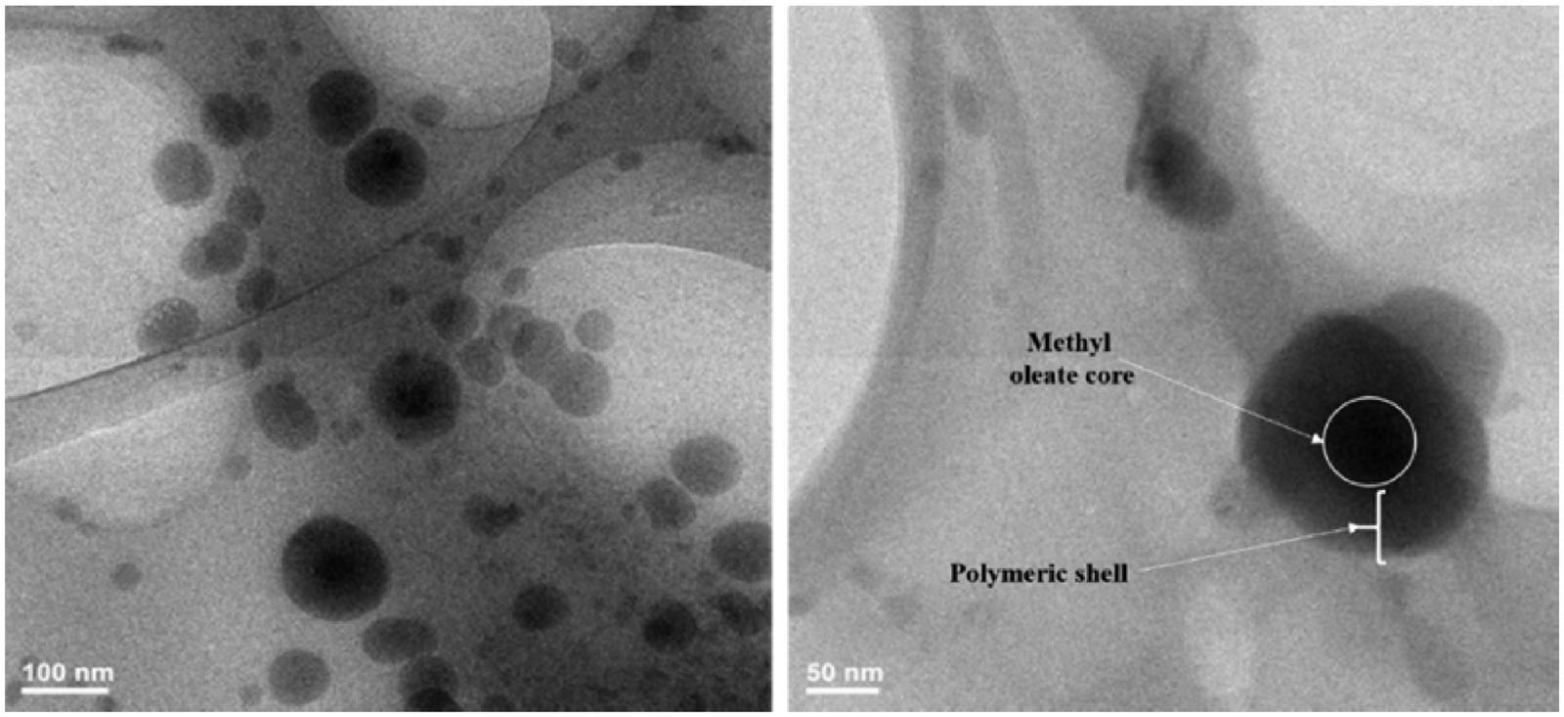
TEM images of nanoparticles with core-shell structure (0.5% PNIPAM/PVA concentration with methyl oleate 0.125% concentration).

TEM images (Figure 10) revealed the expected core-shell structure of PNIPAM/PVA/MO nanoparticles by the presence of a darker area inside the center of the nanoparticles due to the presence of different chemical structures and density which can be attributed to methyl oleate. Thereby, a brighter area was observed alongside the edge of nanoparticles due to the presence of synthetic polymers (PVA and PNIPAM). The methyl oleate core is assumed to occupy about one-third of nanoparticles’ size based on the TEM images. Furthermore, the TEM images confirmed the nanoparticle's size, clear surface, and spherical shape which were previously explained in SEM. Nanoparticles from 5% polymer concentration revealed no core-shell structure probably due to the small dimension unable to embed the methyl oleate.

#### 3.6.2. Morphological characterization of membranes by SEM

**Figure 11.**
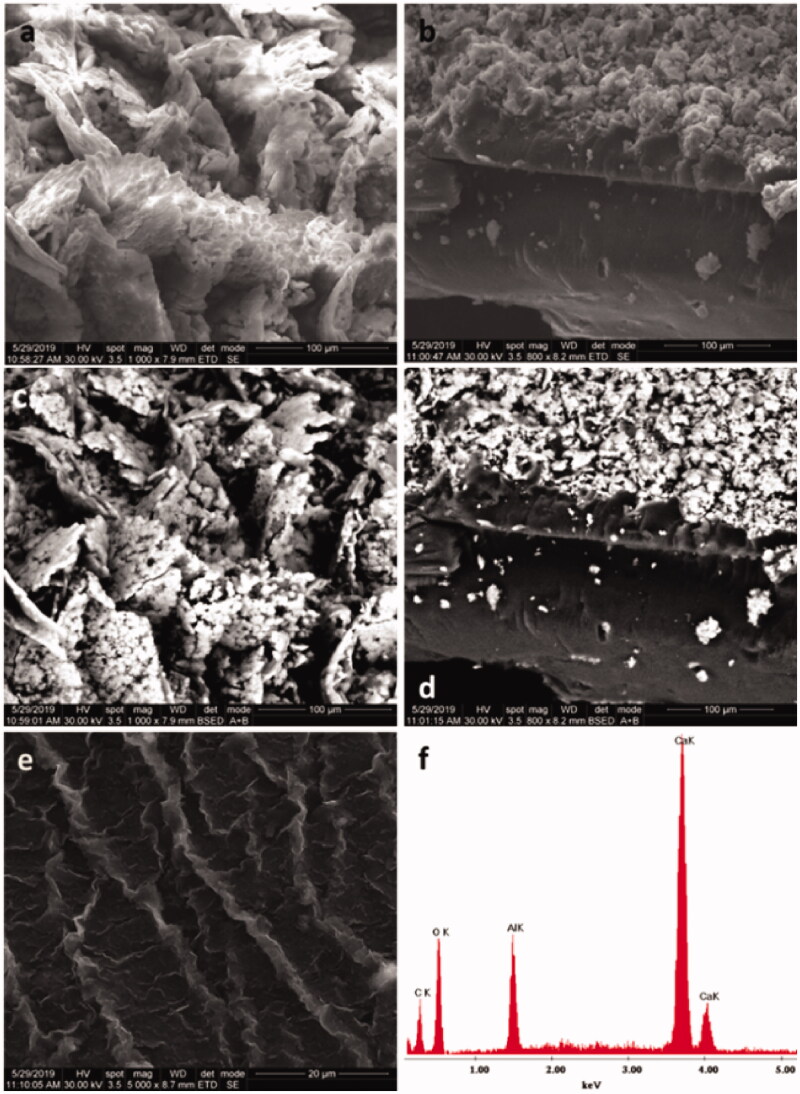
SEM images of: BC/chitosan membrane surface (a,c); lateral view of BC/chitosan; scattering distribution (b,d); crude chitosan surface (e); EDAX elemental analysis (f).

**Scheme 1.**
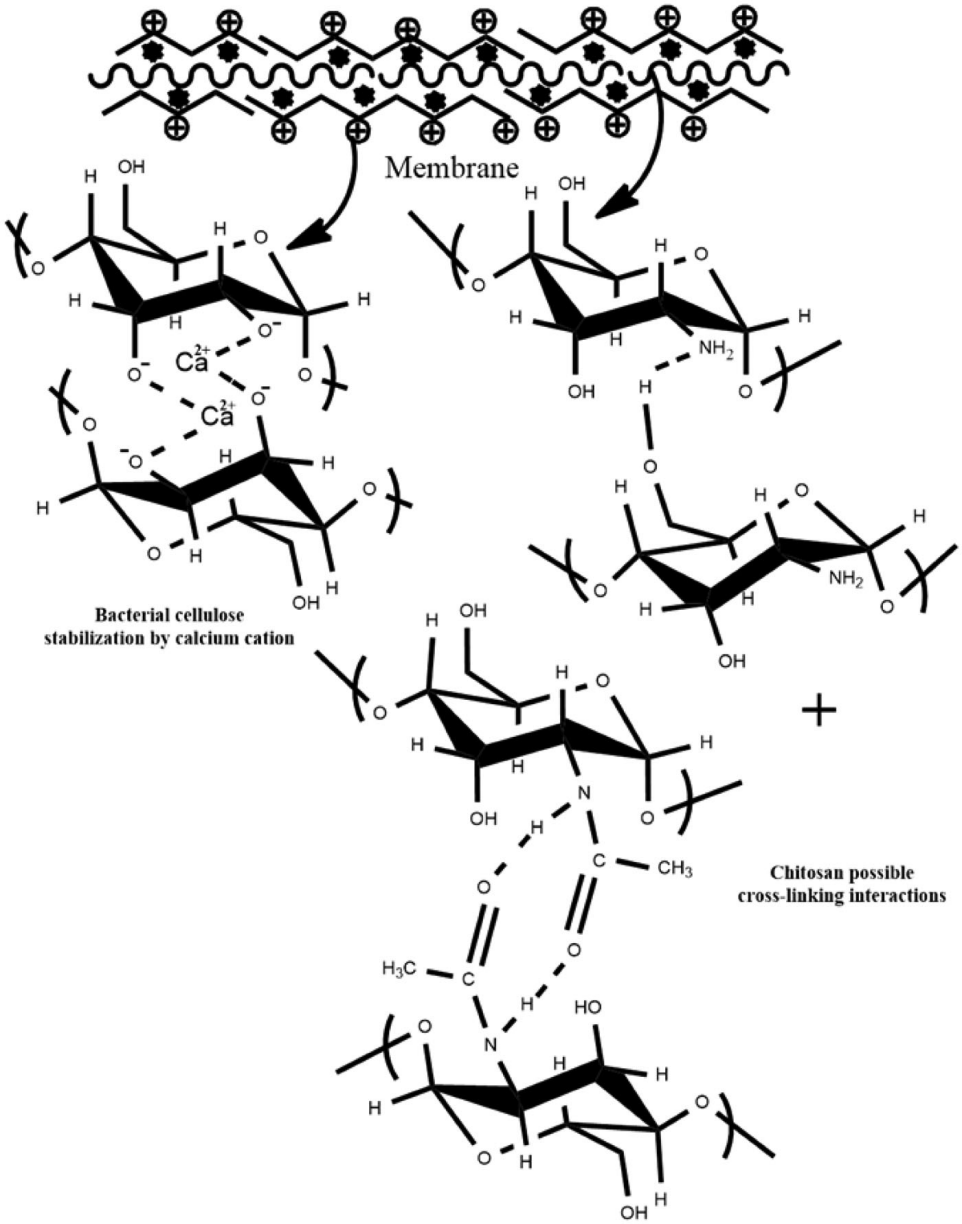
Possible mechanism of physical crosslinking interactions.

SEM images (Figures 11(a,e)) reveal the surface structure and morphology of BC/chitosan and crude chitosan membrane showing the role of every polymer in the membrane. The results highlighted significant differences between surface morphology of BC/chitosan and crude chitosan membrane which suggest a layer structuring of polymers. BC particles seem to dress chitosan, and the compatibility of the two polymers depends on their ratio. A higher chitosan content ensures good compatibility, while a lower ratio of chitosan/BC (15/85—w/w) could lead to polymer separation. Thus, the right ratio between BC and chitosan was chosen in the range 15/85 and 30/70 w/w. Figure 11(b) shows the same layer structuring of the two polymers, BC into the membrane surface and chitosan into the membrane core. Figures 11(c,d) are images in backscattered electrons with the surface distribution of the organic and inorganic components. The images evidence a nice distribution of inorganic calcium components at the membrane surface. The calcium cations could be bound only by the bacterial cellulose suggesting that BC lays at the membrane surface. Furthermore, the EDAX elemental analysis (Figure 11(f)) highlighted the presence of carbon and oxygen elements within polymer and calcium in bacterial cellulose. The presence of Al comes from the sample support of the device. Cellulose is stabilized by ionic interactions between the calcium cations Ca^2+^ and negatively charged alcoholate ions in BC (Scheme 1). The structure and morphology of the novel membrane highlight the possibility to use two polymers that are individually physically/ionically crosslinked, but they act together to design a biocomposite with tunable and desired features. The layered membrane was structured to address several issues. Thus, a BC-calcium layer was shown at the surface for the wound and cells contact while the chitosan core assured the membrane integrity. More, the contact layer showed high biocompatibility while the core layer presented good mechanical properties, swelling capacity, and drug delivery ability.

### 3.7. Dynamic light scattering and zeta potential

#### 3.7.1. Zeta potential

**Figure 12.**
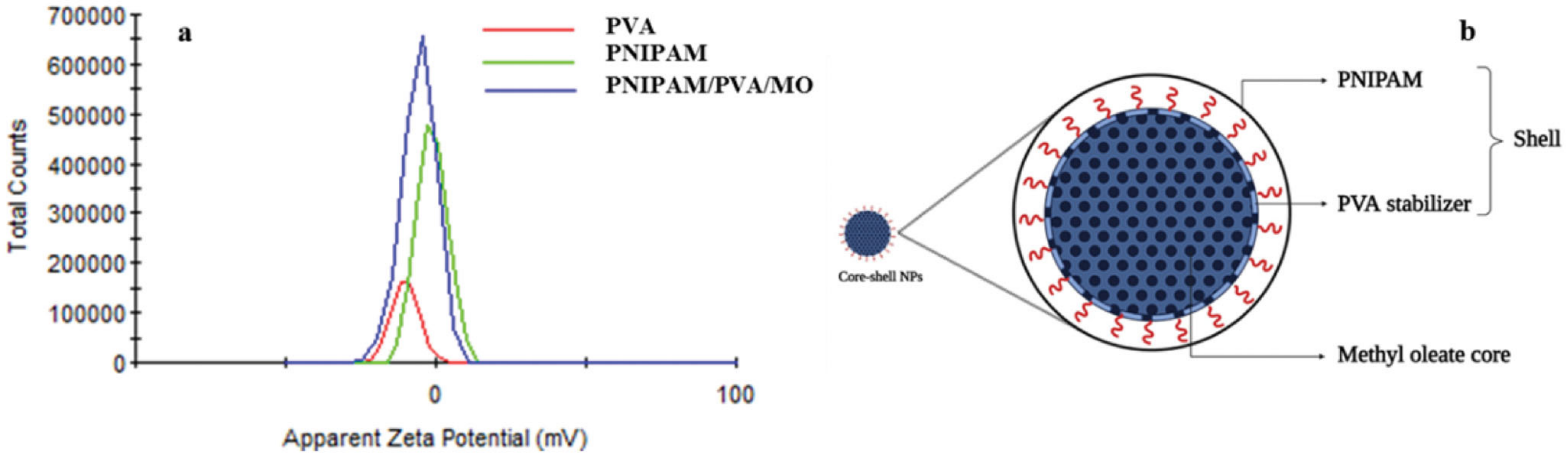
ZETA potential of PVA, PNIPAM, and PNIPAM/PVA/MO nanoparticles (a); Schematic representation of nanoparticles core-shell structure (b).

The ZETA potential investigation (Figure 12(a)) shows the surface charging of PVA, PNIPAM, and PNIPAM/PVA/MO nanoparticles. PVA had a negative zeta potential of about −10.2 mV, PNIPAM −1.28 mV, and nanoparticles of −5.82 mV. ZETA potential results explained the contribution of both PVA and PNIPAM polymers to the surface charging. This result can be explained by the presence of both polymers at the surface of the nanoparticles and not as two distinct polymer layers (Figure 12(b)).

**Scheme 2.**
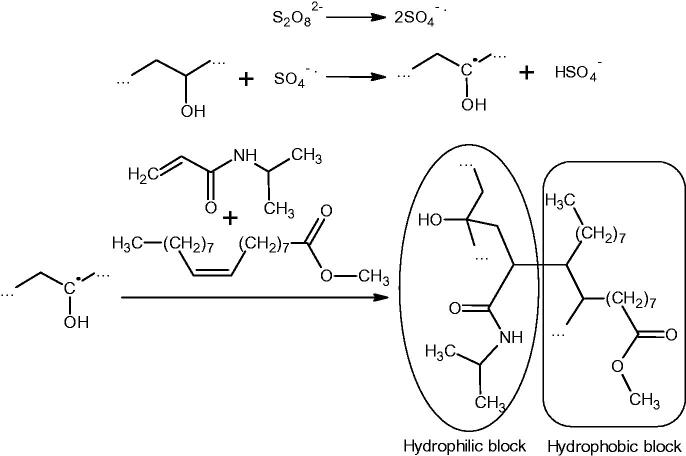
Mechanism of NIPAM grafting to PVA chains.

It is known that persulfates are capable of initiating the crosslinking of polyvinyl alcohol chains by the radical mechanism (Ikada et al., 1974). The radicals formed from polyvinyl alcohol in the presence of *N*-isopropylacrylamide, in turn, are capable of initiating its polymerization. Thus, one can expect the covalent grafting of several *N*-isopropylacrylamide residues to polyvinyl alcohol (Scheme 2). Thus, polyvinyl alcohol is grafted with a copolymer containing not only hydrophilic *N*-isopropylacrylamide residues but also hydrophobic methyl oleate residues. Of course, such a copolymer is capable of self-assembly with the formation of nanoaggregates. This can be the reason for the formation of layers built by chains of both polymers. This nanocarrier with a core-shell structure is a novel approach considering the synergistic effect of shell chemistry (PNIPAM grafting to the PVA backbone) and core part which can also be chemically bound by the shell layer.

#### 3.7.2. Dimensional distribution by DLS

**Figure 13.**
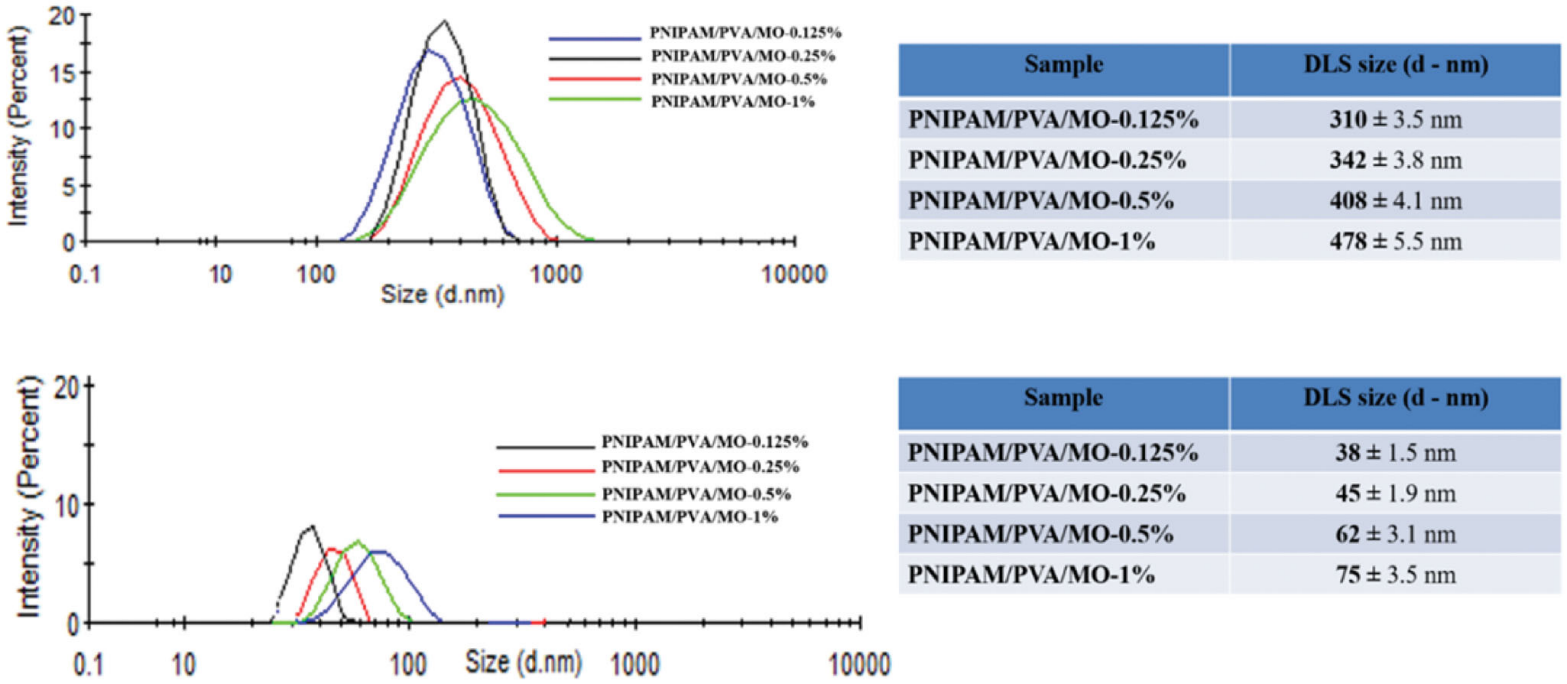
DLS investigation with nanoparticles’ dimensional distribution: nanoparticles from 0.5% PNIPAM/PVA concentration with various methyl oleate concentrations (up); nanoparticles from 5% PNIPAM/PVA concentration with various methyl oleate concentrations (down).

The DLS analysis confirmed the morphological results revealing the important dimensional differences between nanoparticles obtained from 5 and 0.5% polymer concentration. The nanoparticles obtained from 0.5% polymer concentration exhibited sizes in the range 310–478 nm (Figure 13) due to the different amounts of methyl oleate. The size of these nanoparticles increased with the increase of methyl oleate concentration. One may suggest that the organic core of nanoparticles varied with methyl oleate content having a significant influence on the nanoparticle size. Therefore, the high content of methyl oleate led to a bigger nanoparticle size without the formation of smaller size nanoparticles. This could be explained by the fact that a 0.5% polymer concentration could not assure enough PVA stabilizer and PNIPAM to cover the oleate core and to form more smaller size nanoparticles.

The nanoparticles obtained from 5% polymer concentration exhibited a smaller size in the range of 38–75 nm (Figure 13) due to the different amounts of methyl oleate. A larger amount of stabilized PVA stabilizer and PNIPAM led to the decrease of nanoparticle size since there are enough stabilizer and polymer to cover the organic oleate core and form more smaller size nanoparticles. To conclude, the main size parameters are PVA stabilizer and PNIPAM polymer, methyl oleate organic core having smaller influences over nanoparticles size.

### 3.8. In vitro drug release behavior

**Figure 14.**
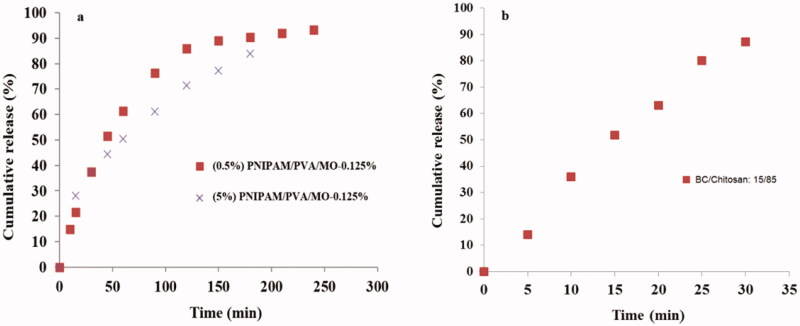
Silver sulfadiazine release profiles for nanoparticles obtained from 0.5 and 5% PNIPAM/PVA concentration (a); drug-loaded BC/chitosan membranes (b).

The release profiles of antibacterial silver sulfadiazine were obtained for different types of carriers: drug-loaded nanoparticles, drug-loaded membranes, and membranes loaded with drug entrapped nanoparticles. First, Figure 14(a) shows the drug release profiles from polymeric nanoparticles. Both nanoparticle formulations (0.5 and 5% polymer concentration) revealed a similar release profile with a faster release in the first 100–120 min followed by a slower release with profile flattening at various efficiency values. The final time release was recorded to be ∼170 min for 0.5% nanoparticles and 240 min for 5% nanoparticles. The release profiles of sulfadiazine-loaded BC/chitosan membranes were also evaluated (Figure 14(b)). In this case, the release profile revealed a very fast release with high efficiency and a short time. High release efficiency of 85–95% in 30 min also revealed a lower capacity to efficiently control the drug release.

**Figure 15.**
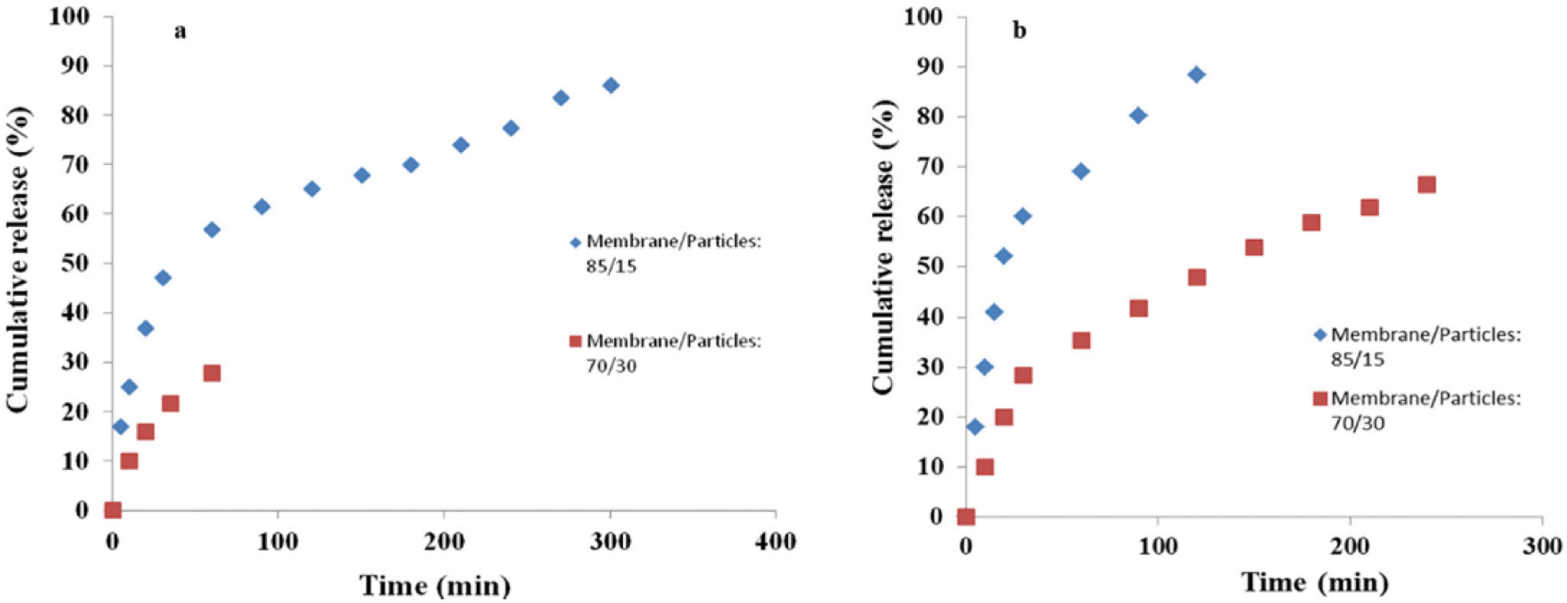
Silver sulfadiazine release profiles for: BC/chitosan membranes loaded with nanoparticles with 0.125% MO (0.5% Polymer concentration) (a); BC/chitosan membranes loaded with nanoparticles with 0.125% MO (5% polymer concentration). The ratios of membrane/nanoparticles were 85/15 and 70/30.

The silver sulfadiazine release profile for membranes loaded with drug entrapped nanoparticles was carried out to improve the control and sustain a prolonged release. The nanoparticles with 0.125% methyl oleate (MO) content were chosen due to the high release efficiency. The higher drug control release profile was studied by varying the BC/chitosan ratio and the ratio of nanoparticles *vs.* BC/chitosan mixture. The membranes loaded with nanoparticles (0.125% MO) obtained from 0.5% polymer concentration were subjected to a drug release test showing different efficiencies. The membranes with a lower amount of drug-loaded nanoparticles expressed higher release efficiency with a release time of about 5 h, and higher control (Figure 15). A higher drug release control as compared to a drug-loaded membrane was expected due to the presence of nanoparticles. Silver sulfadiazine was first released in the support membrane and finally in the medium. The membrane with a higher content of entrapped nanoparticles had a lower drug release efficiency in comparison with the other membranes. In fact, the membranes offered a lower protection and dispersion capacity for the high content of loaded nanoparticles (0.5% polymer concentration). In this case, a high number of bigger size nanoparticles was obtained. Therefore, the drug remains entrapped within nanoparticle agglomerates inside the membrane (Figure 15(a)). Membranes with nanoparticles from 5% polymer concentration were also subjected to a drug release test (Figure 15(b)). Here, the membrane loaded with a lower nanoparticle content showed a faster release profile. This fact can be explained by the good nanoparticles’ dispersion within the support membrane. This favorable dispersion was supported by the low nanoparticles content. Therefore, the membrane assured a lower controlled release over silver sulfadiazine. The increase of the nanoparticles’ content led to the increase of the release time and the decrease of the release efficiency. A more controlled release and the decrease of the release efficiency probably appeared due to the low nanoparticles’ dispersion ability within the membrane. The sulfadiazine is more difficult to be released and remained entrapped within the nanoparticle agglomerates.

### 3.9. PNIPAM/PVA/MO + silver sulfadiazine cytotoxicity screening

**Figure 16.**
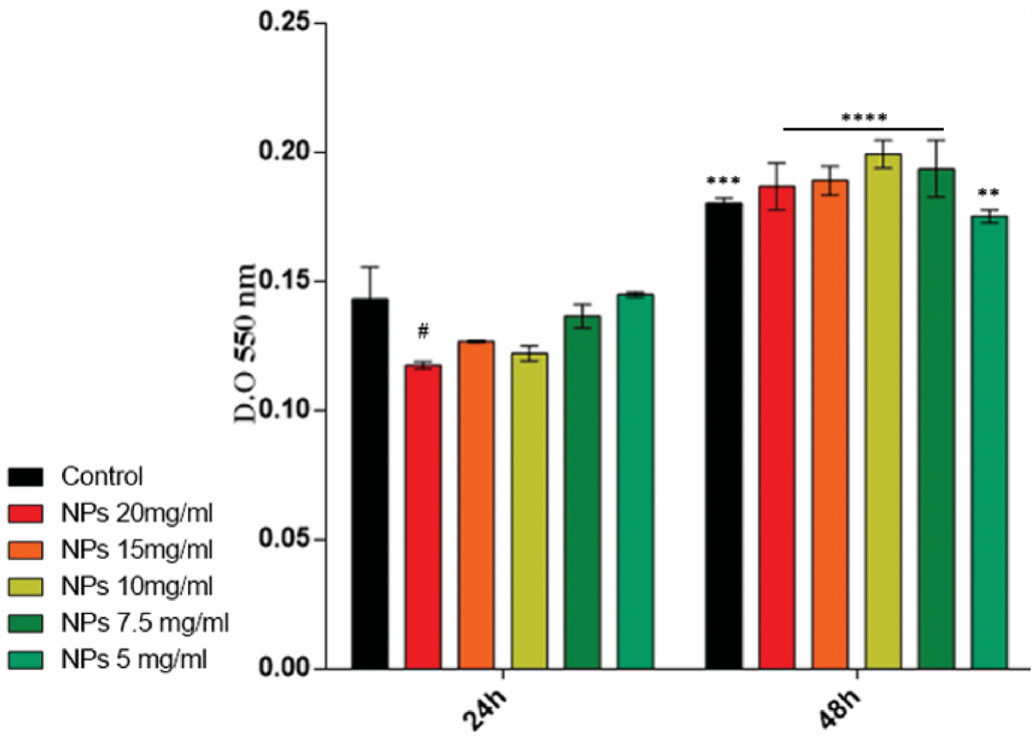
Human dermal fibroblasts viability and proliferation profiles resulting from the MTT assay after 24 and 48 h of culture (NPs = PNIPAM/PVA/MO) (statistical significance: ^#^*p* < .05 20 mg/ml PNIPAM/PVA/MO *vs.* untreated at 24 h; **p < .01 5 mg/ml PNIPAM/PVA/MO at 24 h *vs.* 5 mg/ml PNIPAM/PVA/MO at 48 h; ***p < .001 untreated 24 h *vs.* untreated 48 h; ****p < .0001 7.5, 10, 15, and 20 mg/ml PNIPAM/PVA/MO at 24 h *vs.* 7.5, 10, 15, and 20 mg/ml PNIPAM/PVA/MO at 48 h).

The cell viability and proliferation potential results showed that the majority of the seeded cells displayed good viability both at 24 and 48 h post-seeding when treated with the pristine PNIPAM/PVA/MO (Figure 16). More, after 48 h of culture, a significant increase in cell viability was observed in the samples treated with the pristine PNIPAM/PVA/MO as compared to the previous time point (24 h). These data may suggest that the unloaded microcapsules do not interfere with cell viability and proliferation potential as the treated samples had the same behavior as treated ones.

**Figure 17.**
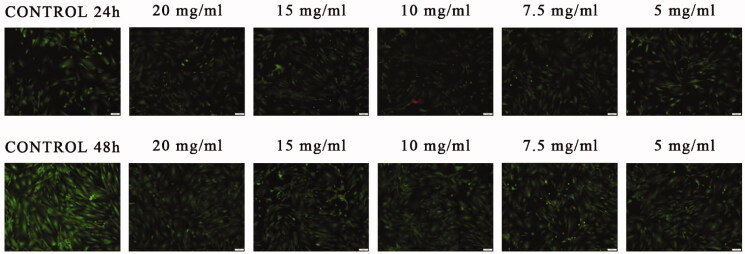
Fluorescence microscopy micrographs of CCD-1070Sk cells stained with calceinAM (green fluorescence) and ethidium bromide (red fluorescence) after 24 and 48 h of treatment with pristine PNIPAM/PVA/MO (NPs = PNIPAM/PVA/MO).

These results are supported by the fluorescence microscopy images obtained after Live/Dead double staining of the cells with calceinAM and ethidium bromide. As shown in Figure 17, all the monolayers treated with the pristine PNIPAM/PVA/MO displayed bright green living cells and few red spots. More, after 48 h of treatment, the cells density was higher as compared with the samples stained after 24 h of treatment, as proof of their growth.

**Figure 18.**
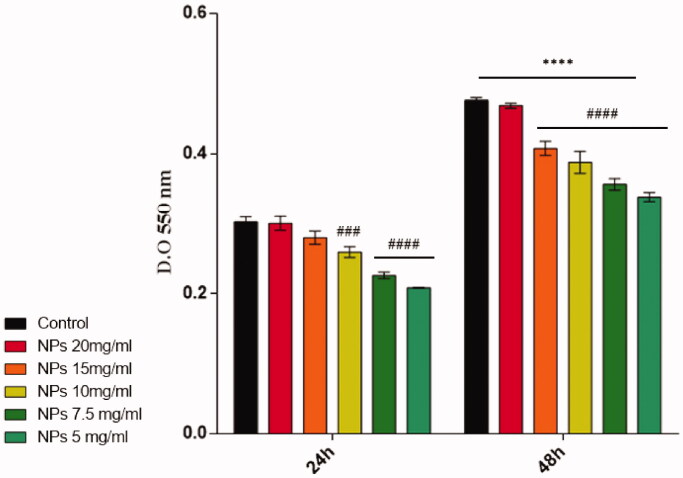
Cytotoxicity evaluation by LDH assay during 48 h of treatment with pristine PNIPAM/PVA/MO (NPs = PNIPAM/PVA/MO) (statistical significance: ^###^*p* < .001 10 mg/ml PNIPAM/PVA/MO *vs.* untreated at 24 h; ^####^*p* < .0001 7.5 and 5 mg/ml PNIPAM/PVA/MO *vs.* untreated at 24 h and ^####^*p* < .0001 15, 10, 7.5, and 5 mg/ml PNIPAM/PVA/MO *vs.* untreated at 48 h; ****p < .0001 untreated, 20, 15, 10, 7.5, and 5 mg/ml PNIPAM/PVA/MO at 24 h *vs.* untreated, 20, 15, 10, 7.5, and 5 mg/ml PNIPAM/PVA/MO at 48 h).

Further, the cytotoxic potential of the PNIPAM/PVA/MO was investigated after performing the LDH assay. The obtained results indicated that the pristine PNIPAM/PVA/MO do not exhibit any cytotoxic effects on the CCD-1070Sk human fibroblast cell line as low levels of LDH activity were found after 24 h of treatment (Figure 18).

### 3.10. Biocompatibility assessment of biocomposites

**Figure 19.**
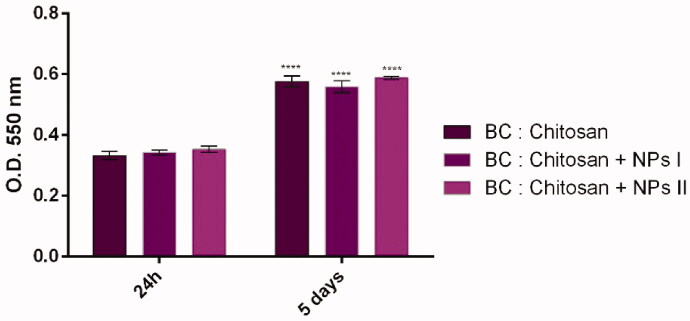
Human dermal fibroblasts viability and proliferation potential on BC/chitosan membranes decorated with silver sulfadiazine loaded PNIPAM/PVA/MO as resulting from the MTT assay after 24 h and 5 days of culture (statistical significance: ****p < .0001, 5 days *vs.* 24 h).

CCD-1070Sk human dermal fibroblasts' cell viability and proliferation potential were investigated in direct contact with the developed BC/chitosan membranes decorated with silver sulfadiazine PNIPAM/PVA/MO. MTT assay was employed after 24 h and 5 days of culture. The spectrophotometric data represented in Figure 19 show that cells survived in contact with all the membranes for 5 days of culture. More, the absorbance of the samples harvested after 5 days of culture was significantly increased as compared with the samples harvested after 24 h of culture, as a sign of cell growth.

## 4. Conclusions

Here, bacterial cellulose/chitosan membranes tailored with silver sulfadiazine loaded PNIPAM/PVA/MO nanoparticles were prepared. Bacterial cellulose/chitosan membranes are formed as a result of the restructuring of the system of intermolecular interactions, which is accompanied by an increase in the density of the network. The increased network density provided a simultaneous decrease in the degree of swelling and an increase in the mechanical strength, as well as the elastic modulus of the bacterial cellulose/chitosan membranes. The composite membranes with a lower amount of drug-loaded polymeric nanoparticles had higher release efficiency and good control due to the good dispersion of the NPs within the BC/chitosan membrane. More, silver sulfadiazine was first released in the support membrane and finally in the medium. The pristine PNIPAM/PVA/MO core-shell polymeric nanoparticles displayed good biocompatibility on CCD-1070Sk human dermal fibroblasts as the treatment with various dilutions did not alter cell viability and proliferation potential as compared with untreated control. We treated CCD-1070Sk cells with 20, 15, 10, 7.5, and 5 mg/ml pristine PNIPAM/PVA/MO core-shell polymeric nanoparticles and after 48 h of exposure, we observed that the cells viability and proliferation potential was not altered by the treatment, suggesting that the new delivery systems display good biocompatibility on human dermal fibroblasts. After this confirmation, we embedded the core-shell polymeric nanoparticles into a biocomposite material composed of bacterial cellulose and chitosan for its prospective use as a wound dressing. Its basic *in vitro* screening revealed good biocompatibility on CCD-1070Sk human dermal fibroblasts in terms of 5 days cell viability. The biocomposite was designed with a layered structure to fulfill the main features of the modern wound dressing in terms of wound protection, moisture, and drug release. The two polymeric networks acting together represent a novel approach that can be more suitable for the next generation of wound dressing materials. Overall, the results presented in the current manuscript indicate that the novel biocomposites are promising candidates for modern dressings for chronic wounds management and could be further developed by *in vivo* validation on animal models.

## References

[CIT0001] Ahmad NH, Mustafa S, Che Man YB. (2015). Microbial polysaccharides and their modification approaches: a review. Int J Food Prop 18:332–47.

[CIT0002] Ahmed J, Gultekinoglu M, Edirisinghe M. (2020). Bacterial cellulose micro-nano fibres for wound healing applications. Biotechnol Adv 41:107549. 32302653 10.1016/j.biotechadv.2020.107549

[CIT0003] Aishah Binti Mohd Isa S, Mohamed R. (2015). Physical and mechanical properties of chitosan and polyethylene blend for food packaging film. Int J Mech Prod Eng 3:51–5.

[CIT0004] Al-Kaabi WJ, Albukhaty S, Al-Fartosy AJM, et al. (2021). Development of *Inula graveolens* (L.) plant extract electrospun/polycaprolactone nanofibers: a novel material for biomedical application. Appl Sci 11:828.

[CIT0005] Almeida IF, Pereira T, Silva NHCS, et al. (2014). Bacterial cellulose membranes as drug delivery systems: an *in vivo* skin compatibility study. Eur J Pharm Biopharm 86:332–6. 23973717 10.1016/j.ejpb.2013.08.008

[CIT0006] Al-Musawi S, Albukhaty S, Al-Karagoly H, et al. (2020). Antibacterial activity of honey/chitosan nanofibers loaded with capsaicin and gold nanoparticles for wound dressing. Molecules 25:4770. 10.3390/molecules25204770 PMC7587596 33080798

[CIT0007] Andrade FK, Costa R, Domingues L, et al. (2010). Improving bacterial cellulose for blood vessel replacement: functionalization with a chimeric protein containing a cellulose-binding module and an adhesion peptide. Acta Biomater 6:4034–41. 20438872 10.1016/j.actbio.2010.04.023

[CIT0008] Asanarong O, Quan VM, Boonrungsiman S, Sukyai P. (2021). Bioactive wound dressing using bacterial cellulose loaded with papain composite: morphology, loading/release and antibacterial properties. Eur Polym J 143:110224.

[CIT0009] Auta GAR, Kwiecien M, Radecka I, Hooley P. (2017). Production and characterization of bacterial cellulose before and after enzymatic hydrolysis. Afr J Biotechnol 16:470–82.

[CIT0010] Bäckdahl H, Risberg B, Gatenholm P. (2011). Observations on bacterial cellulose tube formation for application as vascular graft. Mater Sci Eng C 31:14–21.

[CIT0011] Balasubramani M, Kumar TR, Babu M. (2001). Skin substitutes: a review. Burns 27:534–44. 11451612 10.1016/s0305-4179(01)00018-3

[CIT0012] Bhattarai N, Gunn J, Zhang M. (2010). Chitosan-based hydrogels for controlled, localized drug delivery. Adv Drug Deliv Rev 62:83–99. 19799949 10.1016/j.addr.2009.07.019

[CIT0013] Cerchiara T, Luppi B, Bigucci F, et al. (2002). Physically cross-linked chitosan hydrogels as topical vehicles for hydrophilic drugs. J Pharm Pharmacol 54:1453–9. 12495547 10.1211/00223570281

[CIT0014] Cheng K-C, Catchmark JM, Demirci A. (2009). Enhanced production of bacterial cellulose by using a biofilm reactor and its material property analysis. J Biol Eng 3:12. 19630969 10.1186/1754-1611-3-12 PMC2724407

[CIT0015] Cui F, Li G, Huang J, et al. (2011). Development of chitosan-collagen hydrogel incorporated with lysostaphin (CCHL) burn dressing with anti-methicillin-resistant *Staphylococcus aureus* and promotion wound healing properties. Drug Deliv 18:173–80. 20726806 10.3109/10717544.2010.509363

[CIT0016] Czaja W, Krystynowicz A, Bielecki S, Brown RM. (2006). Microbial cellulose-the natural power to heal wounds. Biomaterials 27:145–51. 16099034 10.1016/j.biomaterials.2005.07.035

[CIT0017] Czaja WK, Young DJ, Kawecki M, Brown RM. (2007). The future prospects of microbial cellulose in biomedical applications. Biomacromolecules 8:1–12. 17206781 10.1021/bm060620d

[CIT0018] Del Prado-Audelo ML, Caballero-Florán IH, Sharifi-Rad J, et al. (2020). Chitosan-decorated nanoparticles for drug delivery. J Drug Delivery Sci Technol 59:101896.

[CIT0019] Derkach SR, Voron’ko NG, Sokolan NI. (2017). The rheology of hydrogels based on chitosan–gelatin (bio)polyelectrolyte complexes. J Dispersion Sci Technol 38:1427–34.

[CIT0020] Dhivya S, Padma VV, Santhini E. (2015). Wound dressings – a review. Biomedicine 5:22. 26615539 10.7603/s40681-015-0022-9 PMC4662938

[CIT0021] Elieh-Ali-Komi D, Hamblin MR. (2016). Chitin and chitosan: production and application of versatile biomedical nanomaterials. Int J Adv Res 4:411–27. PMC5094803 27819009

[CIT0022] Eming SA, Smola H, Krieg T. (2002). Treatment of chronic wounds: state of the art and future concepts. Cells Tissues Organs 172:105–17. 12426487 10.1159/000065611

[CIT0023] Fernandes Queiroz M, Melo KRT, Sabry DA, et al. (2014). Does the use of chitosan contribute to oxalate kidney stone formation? Mar Drugs 13:141–58. 25551781 10.3390/md13010141 PMC4306929

[CIT0024] Fu R, Li C, Yu C, et al. (2016). A novel electrospun membrane based on moxifloxacin hydrochloride/poly(vinyl alcohol)/sodium alginate for antibacterial wound dressings in practical application. Drug Deliv 23:828–9. 24870202 10.3109/10717544.2014.918676

[CIT0025] Fürsatz M, Skog M, Sivlér P, et al. (2018). Functionalization of bacterial cellulose wound dressings with the antimicrobial peptide ε-poly-L-lysine. Biomed Mater 13:025014. 29047451 10.1088/1748-605X/aa9486

[CIT0026] Gadgey K, Sharma G. (2017). Investigation of mechanical properties of chitosan based films: a review. International Journal of Advanced Research in Engineering and Technology (IJARET) 8(6):93–102.

[CIT0027] Gupta NV, Shivakumar HG. (2012). Investigation of swelling behavior and mechanical properties of a pH-sensitive superporous hydrogel composite. Iran J Pharm Res 11:481–93. 24250471 PMC3832170

[CIT0028] Hasan MM, Habib ML, Anwaruzzaman M, et al. (2020). Chapter 3 – processing techniques of chitosan-based interpenetrating polymer networks, gels, blends, composites and nanocomposites. In: Gopi S, Thomas S, Pius A,eds.Handbook of chitin and chitosan,Volume 2: Composites and Nanocomposites from Chitin and Chitosan, Manufacturing and Characterisations, Elsevier, 61-93, 10.1016/C2018-0-03015-7, ISBN: 978-0-12-817968-0.

[CIT0029] He W, Wu J, Xu J, et al. (2020). Bacterial cellulose: functional modification and wound healing applications. Adv Wound Care 10:623–40. 10.1089/wound.2020.1219 PMC8392072 32870775

[CIT0030] Hennink WE, van Nostrum CF. (2002). Novel crosslinking methods to design hydrogels. Adv Drug Deliv Rev 54:13–36. 11755704 10.1016/s0169-409x(01)00240-x

[CIT0031] Hervy M, Santmarti A, Lahtinen P, et al. (2017). Sample geometry dependency on the measured tensile properties of cellulose nanopapers. Mater Des 121:421–9.

[CIT0032] Hospodarova V, Singovszka E, Stevulova N. (2018). Characterization of cellulosic fibers by FTIR spectroscopy for their further implementation to building materials. AJAC 9:303–10.

[CIT0033] Hussain S, Ferguson C. (2006). Best evidence topic report. Silver sulphadiazine cream in burns. Emerg Med J 23:929–32. 17130603 10.1136/emj.2006.043059 PMC2564257

[CIT0034] Iglesias N, Galbis E, Valencia C, et al. (2018). Reversible pH-sensitive chitosan-based hydrogels. Influence of dispersion composition on rheological properties and sustained drug delivery. Polymers 10:392. 10.3390/polym10040392 PMC6415225 30966427

[CIT0035] Ikada Y, Nishizaki Y, Sakurada I. (1974). Reaction of poly(vinyl alcohol) with potassium persulfate and graft copolymerization. J Polym Sci Polym Chem Ed 12:1829–39.

[CIT0036] Jayakumar R, Divya Rani VV, Shalumon KT, et al. (2009). Bioactive and osteoblast cell attachment studies of novel alpha- and beta-chitin membranes for tissue-engineering applications. Int J Biol Macromol 45:260–4. 19523484 10.1016/j.ijbiomac.2009.06.002

[CIT0037] Jayakumar R, Prabaharan M, Sudheesh Kumar PT, et al. (2011). Biomaterials based on chitin and chitosan in wound dressing applications. Biotechnol Adv 29:322–37. 21262336 10.1016/j.biotechadv.2011.01.005

[CIT0038] Jin J, Song M, Hourston DJ. (2004). Novel chitosan-based films cross-linked by genipin with improved physical properties. Biomacromolecules 5:162–8. 14715022 10.1021/bm034286m

[CIT0039] Jóźwiak T, Filipkowska U, Szymczyk P, et al. (2017). Effect of ionic and covalent crosslinking agents on properties of chitosan beads and sorption effectiveness of Reactive Black 5 dye. React Funct Polym 114:58–74.

[CIT0040] Kempe S, Metz H, Bastrop M, et al. (2008). Characterization of thermosensitive chitosan-based hydrogels by rheology and electron paramagnetic resonance spectroscopy. Eur J Pharm Biopharm 68:26–33. 17870449 10.1016/j.ejpb.2007.05.020

[CIT0041] Kevin DB, Bottomley MK, Nixon JS. (1999). Metalloproteinases as targets for anti-inflammatory drugs. 1st ed. Basel: Birkhäuser.

[CIT0042] Kucińska-Lipka J, Gubanska I, Janik H. (2015). Bacterial cellulose in the field of wound healing and regenerative medicine of skin: recent trends and future prospectives. Polym Bull 72:2399–419.

[CIT0043] Kumirska J, Czerwicka M, Kaczyński Z, et al. (2010). Application of spectroscopic methods for structural analysis of chitin and chitosan. Mar Drugs 8:1567–636. 20559489 10.3390/md8051567 PMC2885081

[CIT0044] Ladet SG, Tahiri K, Montembault AS, et al. (2011). Multi-membrane chitosan hydrogels as chondrocytic cell bioreactors. Biomaterials 32:5354–64. 21546080 10.1016/j.biomaterials.2011.04.012

[CIT0045] Li J, Chen W, Wang H, et al. (2009). Preparation of albumin nanospheres loaded with gemcitabine and their cytotoxicity against BXPC-3 cells *in vitro*. Acta Pharmacol Sin 30:1337–43. 19730429 10.1038/aps.2009.125 PMC4007180

[CIT0046] Li M, Liang Y, He J, et al. (2020). Two-pronged strategy of biomechanically active and biochemically multifunctional hydrogel wound dressing to accelerate wound closure and wound healing. Chem Mater 32:9937–53.

[CIT0047] Liang Y, Li Z, Huang Y, et al. (2021). Dual-dynamic-bond cross-linked antibacterial adhesive hydrogel sealants with on-demand removability for post-wound-closure and infected wound healing. ACS Nano 15:7078–93. 33764740 10.1021/acsnano.1c00204

[CIT0048] Liu L, Yao W, Rao Y, et al. (2017). pH-responsive carriers for oral drug delivery: challenges and opportunities of current platforms. Drug Deliv 24:569–81. 28195032 10.1080/10717544.2017.1279238 PMC8241197

[CIT0049] Malcolm Brown R Jr., Saxena IM. (2007). Cellulose: molecular and structural biology. 1st ed. Dordrecht: Springer Netherlands.

[CIT0050] Miles KB, Ball RL, Matthew HWT. (2016). Chitosan films with improved tensile strength and toughness from *N*-acetyl-cysteine mediated disulfide bonds. Carbohydr Polym 139:1–9. 26794940 10.1016/j.carbpol.2015.11.052

[CIT0051] Montembault A, Viton C, Domard A. (2005). Rheometric study of the gelation of chitosan in aqueous solution without cross-linking agent. Biomacromolecules 6:653–62. 15762626 10.1021/bm049593m

[CIT0052] Moscovici M. (2015). Present and future medical applications of microbial exopolysaccharides. Front Microbiol 6:1012. 26483763 10.3389/fmicb.2015.01012 PMC4586455

[CIT0053] Munteanu A, Florescu IP, Nitescu C. (2016). A modern method of treatment: the role of silver dressings in promoting healing and preventing pathological scarring in patients with burn wounds. J Med Life 9:306–15. 27974941 PMC5154321

[CIT0054] Muthu SS, Rathinamoorthy R. (2021). Characteristics of bacterial cellulose, 61-130. In: Bacterial cellulose. Sustainable textiles: production, processing, manufacturing & chemistry. Singapore: Springer.

[CIT0055] Nagahama H, Kashiki T, Nwe N, et al. (2008). Preparation of biodegradable chitin/gelatin membranes with GlcNAc for tissue engineering applications. Carbohydr Polym 73:456–63.

[CIT0056] Nandi S, Winter HH. (2005). Swelling behavior of partially cross-linked polymers: a ternary system. Macromolecules 38:4447–55.

[CIT0057] Nilsen-Nygaard J, Strand SP, Vårum KM, et al. (2015). Chitosan: gels and interfacial properties. Polymers 7:552–79.

[CIT0058] Ong S-Y, Wu J, Moochhala SM, et al. (2008). Development of a chitosan-based wound dressing with improved hemostatic and antimicrobial properties. Biomaterials 29:4323–32. 18708251 10.1016/j.biomaterials.2008.07.034

[CIT0059] Ostrowska-Czubenko J, Pieróg M, Gierszewska-Drużyńska M. (2013). Water state in chemically and physically crosslinked chitosan membranes. J Appl Polym Sci 130:1707–15.

[CIT0060] Peter M, Binulal NS, Soumya S, et al. (2010). Nanocomposite scaffolds of bioactive glass ceramic nanoparticles disseminated chitosan matrix for tissue engineering applications. Carbohydr Polym 79:284–9.

[CIT0061] Portela R, Leal CR, Almeida PL, Sobral RG. (2019). Bacterial cellulose: a versatile biopolymer for wound dressing applications. Microb Biotechnol 12:586–610. 30838788 10.1111/1751-7915.13392 PMC6559198

[CIT0062] Portero A, Teijeiro-Osorio D, Alonso MJ, Remuñán-López C. (2007). Development of chitosan sponges for buccal administration of insulin. Carbohydr Polym 68:617–25.

[CIT0063] Qi Y, Yao X, Du X, An S. (2021). Local anesthetic lidocaine-encapsulated polymyxin-chitosan nanoparticles delivery for wound healing: *in vitro* and *in vivo* tissue regeneration. Drug Deliv 28:285–92. 33501867 10.1080/10717544.2020.1870021 PMC7850372

[CIT0064] Qu J, Zhao X, Liang Y, et al. (2018). Antibacterial adhesive injectable hydrogels with rapid self-healing, extensibility and compressibility as wound dressing for joints skin wound healing. Biomaterials 183:185–99. 30172244 10.1016/j.biomaterials.2018.08.044

[CIT0065] Radu IC, Hudita A, Zaharia C, et al. (2019). Poly(3-hydroxybutyrate-CO-3-hydroxyvalerate) PHBHV biocompatible nanocarriers for 5-FU delivery targeting colorectal cancer. Drug Deliv 26:318–27. 30896267 10.1080/10717544.2019.1582729 PMC6442118

[CIT0066] Radwan-Pragłowska J, Piątkowski M, Deineka V, et al. (2019). Chitosan-based bioactive hemostatic agents with antibacterial properties-synthesis and characterization. Molecules 24:2629. 10.3390/molecules24142629 PMC6681126 31330957

[CIT0067] Rinaudo M. (2006). Chitin and chitosan: properties and applications. Prog Polym Sci 31:603–32.

[CIT0068] Robson MC, Steed DL, Franz MG. (2001). Wound healing: biologic features and approaches to maximize healing trajectories. Curr Probl Surg 38:72–140. 11452260 10.1067/msg.2001.111167

[CIT0069] Ryu JH, Lee Y, Kong WH, et al. (2011). Catechol-functionalized chitosan/pluronic hydrogels for tissue adhesives and hemostatic materials. Biomacromolecules 12:2653–9. 21599012 10.1021/bm200464x

[CIT0070] Scherner M, Reutter S, Klemm D, et al. (2014). *In vivo* application of tissue-engineered blood vessels of bacterial cellulose as small arterial substitutes: proof of concept? J Surg Res 189:340–7. 24726059 10.1016/j.jss.2014.02.011

[CIT0071] Singh DK, Ray AR. (2000). Biomedical applications of chitin, chitosan, and their derivatives. J Macromol Sci C 40:69–83.

[CIT0072] Sulaeva I, Henniges U, Rosenau T, Potthast A. (2015). Bacterial cellulose as a material for wound treatment: properties and modifications. A review. Biotechnol Adv 33:1547–71. 26253857 10.1016/j.biotechadv.2015.07.009

[CIT0073] Tsouko E, Kourmentza C, Ladakis D, et al. (2015). Bacterial cellulose production from industrial waste and by-product streams. Int J Mol Sci 16:14832–49. 26140376 10.3390/ijms160714832 PMC4519874

[CIT0074] Ul-Islam M, Khan T, Park JK. (2012). Nanoreinforced bacterial cellulose-montmorillonite composites for biomedical applications. Carbohydr Polym 89:1189–97. 24750931 10.1016/j.carbpol.2012.03.093

[CIT0075] Wen X, Zheng Y, Wu J, et al. (2015). *In vitro* and *in vivo* investigation of bacterial cellulose dressing containing uniform silver sulfadiazine nanoparticles for burn wound healing. Progr Nat Sci Mater Int 25:197–203.

[CIT0076] Weng L, Chen X, Chen W. (2007). Rheological characterization of *in situ* crosslinkable hydrogels formulated from oxidized dextran and *N*-carboxyethyl chitosan. Biomacromolecules 8:1109–15. 17358076 10.1021/bm0610065 PMC2572577

[CIT0077] Weyell P, Beekmann U, Küpper C, et al. (2019). Tailor-made material characteristics of bacterial cellulose for drug delivery applications in dentistry. Carbohydr Polym 207:1–10. 30599988 10.1016/j.carbpol.2018.11.061

[CIT0078] Wong RSH, Ashton M, Dodou K. (2015). Effect of crosslinking agent concentration on the properties of unmedicated hydrogels. Pharmaceutics 7:305–19. 26371031 10.3390/pharmaceutics7030305 PMC4588202

[CIT0079] Zaharia BG, Vasile CE, Bunea MC, Casarica A, et al. (2014). Bacterial cellulose-polyhydroxyalkanoates composites synthesis, physico-chemical characterization and biological evaluation for tissue engineering. Mater Plast 51:1–5.

[CIT0080] Zając A, Hanuza J, Wandas M, Dymińska L. (2015). Determination of *N*-acetylation degree in chitosan using Raman spectroscopy. Spectrochim Acta A Mol Biomol Spectrosc 134:114–20. 25011040 10.1016/j.saa.2014.06.071

[CIT0081] Zhang B, He J, Shi M, et al. (2020). Injectable self-healing supramolecular hydrogels with conductivity and photo-thermal antibacterial activity to enhance complete skin regeneration. Chem Eng J 400:125994.

[CIT0082] Zhao D, Yu S, Sun B, et al. (2018). Biomedical applications of chitosan and its derivative nanoparticles. Polymers 10:462. 10.3390/polym10040462 PMC6415442 30966497

[CIT0083] Zhao X, Liang Y, Guo B, et al. (2021). Injectable dry cryogels with excellent blood-sucking expansion and blood clotting to cease hemorrhage for lethal deep-wounds, coagulopathy and tissue regeneration. Chem Eng J 403:126329.

[CIT0084] Zheng L, Li S, Luo J, Wang X. (2020). Latest advances on bacterial cellulose-based antibacterial materials as wound dressings. Front Bioeng Biotechnol 8:593768. 33330424 10.3389/fbioe.2020.593768 PMC7732461

